# Framework Nucleic Acids as a Nanocontainer for Delivering Heterogeneous Molecular Drugs

**DOI:** 10.3390/pharmaceutics18040439

**Published:** 2026-04-01

**Authors:** Miao Yang, Xiufan Lou, Jiahong Song, Jia Wang, Hongzhen Peng, Lihua Wang

**Affiliations:** Institute of Materiobiology, College of Sciences, Shanghai University, Shanghai 200444, China; 17837702067@shu.edu.cn (M.Y.); louxiufan@shu.edu.cn (X.L.); songjh@shu.edu.cn (J.S.); wangjia124@shu.edu.cn (J.W.)

**Keywords:** framework nucleic acid, DNA nanostructure, drug delivery, nanocontainer, structure–activity relationship

## Abstract

Framework nucleic acids (FNAs) are a class of nucleic acid-based nanostructures characterized by their unique precise structures, excellent biocompatibility and stability, robust loading capacity, and distinctive distribution and metabolic behaviors. They are widely applied in frontier fields such as nanodevices, biosensing, and drug delivery. In recent years, research on FNAs has gradually developed from the design and synthesis of nucleic acid nanostructures to practical applications, particularly in providing precise nanocontainers for heterogeneous molecular drugs such as small molecules, peptides, and proteins. Acting as a drug delivery system, FNA nanocontainers could be utilized to address multiple issues inherent in the application of heterogeneous molecular drugs, including hydrophobicity, affinity, and stability. However, they also face challenges such as low drug carrier capacity, potential immunogenicity, and insufficient long-term stability in vivo, necessitating the development of new strategies. This article focuses on composite drugs of small molecules, peptides, and proteins carried by FNAs, elucidates the design principles of FNA carriers, the interaction modes between FNAs and drug molecules, and the physicochemical properties and biological effects/efficacy of FNA–drug complexes, and summarizes the structure–activity relationship patterns. Furthermore, obstacles limiting clinical transformation are proposed to provide beneficial suggestions for the future development of FNA-based drugs.

## 1. Introduction

Research in modern drug development primarily encompasses categories such as small molecule drugs, peptide drugs, and protein drugs, each with unique advantages and applicable scopes. Small molecule drugs, characterized by their low molecular weight, relatively simple synthesis, and suitability for oral administration, are the most widely used in disease treatment. Peptide drugs, as “short-chain amino acids” bridging small molecules and proteins, demonstrate significant potential in areas like oncology due to their high targeting specificity, low toxicity, and potent activity. Protein drugs, such as antibodies, classified as macromolecular biologics, exhibit exceptional target specificity and affinity, playing a crucial role in tumor immunotherapy and autoimmune diseases. These three categories collectively form the cornerstone of modern drug development, providing diverse therapeutic solutions for various diseases through distinct mechanisms of action. However, in clinical practice, multiple factors still constrain their efficacy. For instance, small molecule drugs often exhibit low solubility [1], non-specific distribution [2], and susceptibility to drug resistance [3] and systemic toxicity [4]. Similarly, peptide and protein drugs frequently demonstrate weak anti-enzymatic degradation capabilities and difficulty penetrating cell membranes [5], meaning that further advancements are necessary in carrier technology for further optimization.

Although various delivery systems such as lipid nanoparticles (LNPs) [6,7], polymers [8,9], porous metal–organic frameworks [10], inorganic nanomaterials [11], and biomimetic carriers [12] have been used to improve drug stability and in vivo distribution, they still possess significant limitations in terms of structural controllability, long-term stability, and biosafety. From the perspective of delivery efficiency, LNPs demonstrate high encapsulation efficiency (approximately 80–95%) and certain transfection capacity; however, their extremely low endocytic escape efficiency, coupled with susceptibility to drug leakage or uncontrolled release, leads to a substantial decline in final delivery efficiency. Porous metal–organic frameworks possess advantages in high drug loading capacity but still face challenges in achieving controlled release while maintaining high loading levels [10]. Inorganic nanomaterials struggle to fully realize their delivery efficiency due to insufficient in vivo stability and susceptibility to clearance by the reticuloendothelial system [11]. As for biomimetic carriers, despite their excellent immune evasion and targeted enrichment capabilities, they encounter limitations in drug delivery methods that hinder stable control of overall delivery efficiency [12].

In recent years, nucleic acid nanostructures have been widely applied in the field of drug delivery, particularly those with well-defined frameworks that offer optimal accommodation space. Framework nucleic acids (FNAs) are nanostructures with specific shapes and sizes self-assembled from nucleic acid molecules based on the principle of complementary base pairing [13]. Due to the excellent monodispersity, high programmability, and good addressability, they have demonstrated extensive application potential across multiple disciplines [14,15,16,17,18]. In drug delivery systems, FNAs, as structures composed of natural biological macromolecules, also possess characteristics such as high biocompatibility and a strong ability to overcome biological barriers [19,20]. Research utilizing FNA structures for delivering nucleic acid-based drugs, small molecule drugs, peptide drugs, and protein-based drugs has emerged as a research hotspot, particularly offering novel approaches for the delivery of heterogeneous molecular drugs. The delivery of such drugs involves complex interactions between carriers and drugs, significantly influencing drug properties and biological behavior.

**Figure 1:**
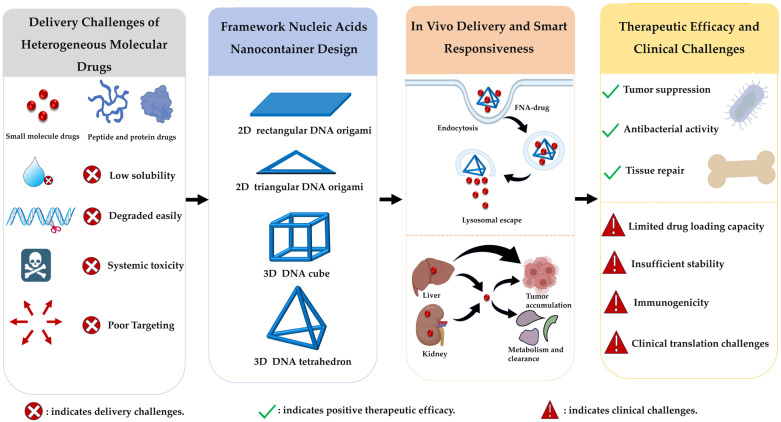
FNA-based delivery systems for heterogeneous molecular drugs.

Rapid advancements have been achieved in FNA research for drug delivery applications; however, existing reviews predominantly focus on their applications in biosensing or nucleic acid-based drug delivery systems. There remains a lack of systematic theoretical analysis and pattern identification regarding FNAs as ideal nanocarriers for non-nucleic acid drug delivery, particularly in molecular-level studies of their structural design, drug loading mechanisms, and structure–activity relationships with pharmaceutical molecules. Leveraging the structural advantages of FNAs to achieve efficient drug loading, controlled release, and precise targeting—thereby enhancing therapeutic efficacy in complex diseases—remains a critical scientific challenge requiring further resolution. Therefore, a comprehensive review of current research progress and underlying principles in this field not only addresses gaps in the existing literature and clarifies the strengths and limitations of different strategies but also provides a theoretical foundation for advancing FNA delivery systems toward clinical translation. Based on this issue, this article provides a comprehensive review of the delivery of non-nucleic acid drugs. This review will focus on the design principles of FNAs, followed by the interaction modes between FNAs and drugs, and recent progress in their application as nanocontainers for carrying heterogeneous molecular drugs. The structure–activity relationship patterns of FNAs and heterogeneous molecular drugs will also be described in a later section following detailed examples, and an objective analysis of potential problems in clinical transformation will be provided, offering references and outlooks for the development of next-generation FNA-drug delivery systems (Figure 1).

## 2. Design Principles and Drug Interaction Modes of FNA Nanocontainers

Generally, drug molecules are loaded onto delivery vehicles due to their interactions with these nanocontainers. Different nanocontainers often exhibit distinct interaction patterns, and the interaction patterns between different drug molecules and the same type of nanocontainer can also vary. Therefore, understanding the inherent properties of the drug itself and the characteristics of the nanocontainer is essential for comprehending the mechanisms of drug delivery systems.

### 2.1. Physicochemical Properties and Delivery Requirements of Heterogeneous Molecular Drugs

Heterogeneous molecular drugs are therapeutic agents whose efficacy does not rely on nucleic acid sequence complementarity, including small molecule drugs, peptides drug and protein-based macromolecular drugs, which exhibit fundamentally different structures and properties compared to FNA nanocontainers. These differences are also reflected in the structural diversity of drug molecules and their delivery requirements for various diseases. For instance, small molecule drugs primarily address critical challenges such as solubility and targeting, while protein drugs require stability optimization. Transdermal drugs focus on enhancing permeability, whereas oncology therapies demand comprehensive regulation of distribution and metabolism. Consequently, the structural design and interaction patterns of FNA nanocontainers must be specifically engineered for each drug class.

#### 2.1.1. Small Molecule Drugs

Small molecule drugs usually refer to compounds with clear chemical structures and a molecular weight of less than 500 Da. In the huge family of small molecule drugs, chemotherapeutic drugs are one of the classes gaining widespread attention. Since the first small molecule antitumor drug targeting tyrosine kinase, imatinib, was approved by the U.S. Food and Drug Administration (FDA), small molecule antitumor drugs have developed rapidly. Therapeutic small molecule chemotherapeutic drugs with different mechanisms of action and structural characteristics have been continuously applied in the treatment of malignant tumors. Common small molecule chemotherapeutic drugs include doxorubicin [21] and inhibitor drugs like paclitaxel [22]; however, these drugs often suffer from low bioavailability, poor water solubility, and side effects such as cardiotoxicity, neurotoxicity, and bone marrow suppression (Table 1).

Metal complex drugs refer to a class of theranostic drugs with metal elements as the structural core. Their essential feature lies in utilizing the unique coordination geometry, redox activity, and spatial structural characteristics of the metal center to form coordinate covalent bonds or participate in electron transfer processes, thereby achieving precise binding to and intervention of specific biological targets. Metal complexes drugs, such as platinum complexes [23], have played a significant role in anticancer therapy, antibacterial applications, and diagnostic imaging [24]. However, these metal drugs also have certain limitations, such as complex in vivo metabolic processes, potential toxicity risks, and poor stability in biological samples (Table 1). In addition, antimetabolite drugs also act as a class of molecular cargo loaded into FNA nanocontainers (Table 1).

Photosensitizers are functional molecules that can absorb photons of specific wavelengths and transition to an excited state, then transfer the acquired energy or electrons to surrounding reactants via energy or electron transfer, inducing a series of photochemical or photobiological reactions. Since light can be precisely controlled in time and space, photosensitizers exhibit significant spatiotemporal controllability in biomedical applications such as photodynamic therapy. Consequently, photosensitizers have been widely used in research for treating diseases like cancer [25]. Commonly used functional photosensitizers include IR780 and BMEPC. However, traditional photosensitizers also face challenges such as poor water solubility and weak targeting (Table 1). In addition, FNAs can load various small molecule drugs as treatment strategies for a variety of diseases (Table 1).

#### 2.1.2. Peptide and Protein Drugs

Peptides are biologically active polymer compounds formed by amino acids connected by peptide bonds. Drugs with more than 100 amino acids are classified as protein drugs, while those with fewer than 100 are peptide drugs [26]. Compared to small molecule drugs, peptide and protein drugs have better biocompatibility and are less prone to accumulation in the body. However, the physicochemical properties of these drugs are unstable, with short half-lives, susceptibility to clearance by the body, and poor cell permeability (Table 1) [27].

Table 1 lists categories of non-nucleic acid drug molecules delivered via FNA nanocarriers, their therapeutic mechanisms, and current limitations of single-agent use. The small molecule drugs listed are primarily employed for tumor treatment. Anthracycline compounds are extensively utilized in treating various hematologic malignancies by inserting into DNA double helix base pairs. Inhibitor-based drugs (e.g., paclitaxel) target tumors such as breast and ovarian cancers by inhibiting mitosis or kinase activity in rapidly proliferating cancer cells. Metal complexes leverage DNA-damaging and reactive oxygen species-generating capabilities to treat tumors including cervical cancer, ovarian cancer, and lymphoma. Antineoplastic agents (e.g., gemcitabine) induce apoptosis by suppressing DNA synthesis, effectively managing pancreatic cancer and non-small-cell lung cancer. Photosensitizers are mainly utilized in photodynamic therapy for cancer. Additionally, naturally derived molecules (e.g., quercetin) exhibit broad pharmacological effects encompassing anti-inflammatory, anticancer, antioxidant, and immunomodulatory activities. As for peptide- and protein-based drugs, antimicrobial peptides (e.g., GL13K and His-5) combat bacterial infections by disrupting bacterial membrane integrity, while antitumor peptides (e.g., KLA) target mitochondrial function in tumor therapy. Healing-promoting peptides are primarily used for tissue regeneration and angiogenesis-related treatments. Protein-based drugs, including enzymes, antibodies, and cytokines, are widely applied in cancer therapy, metabolic regulation, and immunotherapy.

In summary, although heterogeneous molecular drugs possess significant biological activities, their in vivo application remains limited by issues such as insufficient water solubility, uncontrollable biodistribution and retention behavior, and potential toxicity risks. There is an urgent need to develop an in vivo delivery system that combines good biocompatibility with targeted delivery capabilities.

### 2.2. FNAs as Nanocontainers for Drug Delivery

DNA is not only a carrier of genetic information; its highly programmable molecular structure also provides a unique foundation for precise nanometer-scale construction. DNA nanotechnology, developed based on the principle of Watson–Crick base pairing, enables researchers to construct DNA nanostructures with defined geometries and spatial scales in a highly predictable manner. These structures show significant advantages in structural regularity, addressable chemical modification, and spatial designability [28]. Based on this research foundation, Fan et al. [13] proposed the concept of “framework nucleic acids (FNAs)” in 2018 to describe a class of nucleic acid nanostructures formed by bottom-up self-assembly with clear morphology and size. The following part of this chapter will provide an overview of the structural characteristics of different types of FNAs and their construction strategies, and briefly describe their interactions with drugs loaded in the nanocontainer.

#### 2.2.1. Design Principles of Framework Nucleic Acids

DNA nanotechnology was first proposed by Professor Seeman in 1982 [29]. This technology can construct DNA nanostructures of different sizes, dimensions, and shapes. Commonly used FNAs are generally divided into DNA origami structures [30] and DNA polyhedral structures [31], which have shown broad application prospects in fields such as biosensing and imaging, targeted drug delivery, early disease diagnosis and treatment, and regenerative medicine.

**Figure 2:**
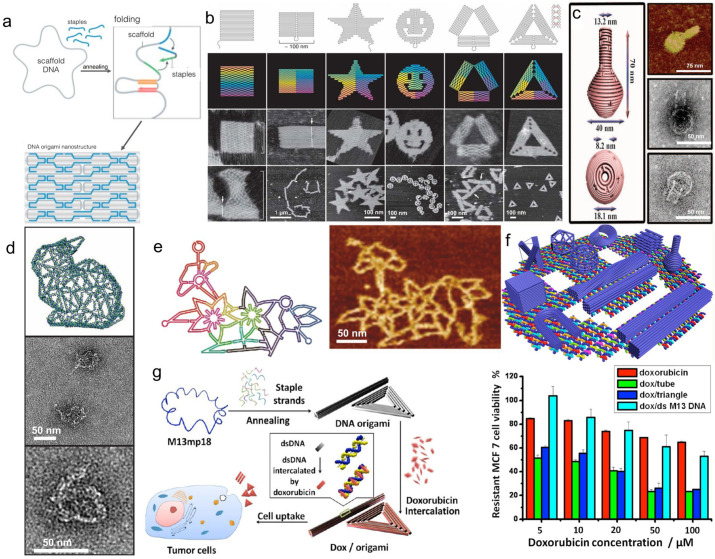
(**a**) Basic principles of DNA origami technology. DNA origami is constructed by mixing a long scaffold strand with numerous short staple strands, where sequence-specific base pairing drives their hybridization, thereby directing the scaffold to fold into a predefined nanostructure. Reprinted with permission from Ref. [32]. Copyright © 2022, The Authors. (**b**) DNA origami images, including square, rectangular, star, smiley face, triangle with rectangular regions, and acute-angled triangle. The images in (**b**) without scale bars are presented at a size of 165 nm × 165 nm. Reprinted with permission from Ref. [33]. Copyright © 2022, The Authors. (**c**) Structural schematic of a DNA nano-vase and its atomic force microscopy (AFM) images and transmission electron microscopy (TEM) images. Reprinted with permission from Ref. [34]. Copyright © 2011, The American Association for the Advancement of Science. (**d**) Frontal view of a complete DNA origami rabbit pattern and its TEM images. Scale bars are 50 nm. Reprinted with permission from Ref. [36]. Copyright © 2023, Wiley-VCH. (**e**) Wireframe structure of a DNA origami nanoflower and its AFM images. Reprinted with permission from Ref. [37]. Copyright © 2017, American Chemical Society. (**f**) Different DNA origami structural models. Reprinted with permission from Ref. [37]. Copyright © 2017, American Chemical Society. (**g**) Schematic diagram of triangular and tubular DNA origami structures for doxorubicin delivery, and the effect of doxorubicin-loaded DNA origami structures on the survival rate of the drug-resistant breast cancer cell line MCF 7. Reprinted with permission from Ref. [41]. Copyright © 2012, American Chemical Society.

DNA origami technology, first proposed by Rothemund in 2006 [29], is a powerful bottom-up self-assembly technique. It uses a long strand of viral DNA as a “scaffold strand” and folds it into complex 2D or 3D nanostructures through a specific synthesis procedure, using short DNA strands complementary to specific regions of the scaffold strand as “staple strands”. This allows precise control of geometry and flexible construction of complex structures to build 2D or 3D nanostructures (Figure 2a) [32], such as star and rectangular structures (Figure 2b) [33], vase (Figure 2c) [34], rabbit (Figure 2d) [35,36], flower (Figure 2e) [37], map of China [38], and boxes (Figure 2f) [39]. Utilizing the spatial addressability, high programmability, design flexibility, and structural stability of DNA origami, these nanostructures have been widely used for molecular drug delivery, partially addressing the limited therapeutic effects of traditional chemotherapeutic drugs caused by drug resistance [40]. For example, in 2012, Jiang et al. [41] used DNA origami to construct 2D triangular and 3D tubular nanocarriers for doxorubicin (Dox) delivery, achieving a loading efficiency of 50–60% after 24-h incubation, which is significantly better than that of unstructured double-stranded M13 RF I DNA (Figure 2g). When the Dox concentration was raised to 100 μM, compared with free Dox and drug-loaded double-stranded DNA, drug-loaded DNA origami could induce cell death in drug-resistant breast cancer cell lines, increase the uptake of Dox by drug-resistant cells, and reverse drug resistance.

**Figure 3:**
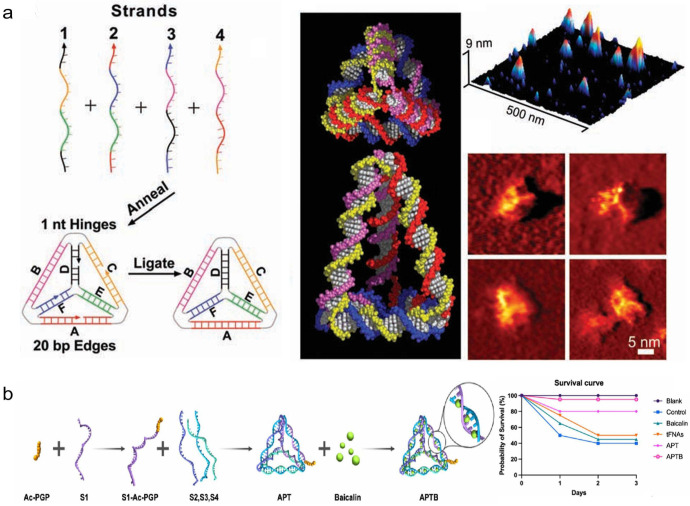
(**a**) Schematic diagram of the design, synthesis and characterization of DNA tetrahedra. The left figure shows the assembly process of DNA tetrahedra, where four different oligonucleotide strands anneal to form a DNA tetrahedron with an edge length of 20 bp. The middle figure shows the space-filling models of DNA tetrahedra with edge lengths of 20 bp and 30 bp, where each color represents an oligonucleotide strand. The right figure is the atomic force microscopy (AFM) characterization image: the top image shows multiple DNA tetrahedra with uniform height distributed on the mica surface, and the bottom image presents a high-resolution AFM image of four DNA tetrahedra. Reprinted with permission from Ref. [42]. Copyright © 2005, The American Association for the Advancement of Science. (**b**) Schematic diagram of APTB synthesis and its therapeutic effects on septic mice. Reprinted with permission from Ref. [46]. Copyright © 2025, American Chemical Society.

DNA polyhedrons, as a class of 3D self-assembled nanostructures with regular geometries, offer significant advantages such as precise structural control, excellent biocompatibility, and ease of surface functionalization. The tetrahedron framework nucleic acid (tFNA) is one of the simplest 3D DNA polyhedral structures, formed by four precisely designed single-stranded DNA sequences that are partially complementary and self-assemble through annealing under specific synthesis protocols (Figure 3a) [42]. This structure features a highly symmetrical 3D framework, high synthesis yield, and good biocompatibility, allowing rapid cell entry without transfection reagents while maintaining structural integrity in complex biological environments [43]. It has been widely applied in targeted therapy for various complex diseases such as osteoarthritis [44], idiopathic pulmonary fibrosis [45], systemic inflammation [46], osteoporosis [47], chronic kidney disease [48], and ischemic stroke [49], showing significant clinical transformation potential. tFNAs, through their clear spatial confinement effects and controllable molecular interaction patterns, have improved issues like low encapsulation efficiency and insufficient cellular uptake of traditional hydrophobic drug molecules to some extent. Bai et al. [47] designed a tFNAs/curcumin complex to solve the problems of poor water solubility, rapid metabolism, and low cellular uptake efficiency of curcumin (Cur), encapsulating Cur in the inner cavity of the framework through trench binding and electrostatic actions. Studies have shown that the encapsulation rate can reach more than 80% at a tFNA/Cur concentration of 40 μM. More importantly, tFNAs significantly extended the retention time of Cur in tibial bone defect areas and improved cellular uptake efficiency. In an osteoporotic rat model, the bone formation ability of the tFNAs/Cur group was better than that of the Cur group alone, indicating that the spatial confinement effect of FNAs can effectively improve the delivery efficiency of hydrophobic drugs, providing a new idea for bone regeneration therapy. Zhou et al. [46] further expanded the application of tFNAs by constructing an Ac-PGP-tFNA (APT) system that recognizes CXCR2 receptors on the surface of neutrophils by modifying the targeted peptide Ac-PGP at the apex of tFNA through click chemistry and using hydrophobic actions to load baicalin. In this design, the particle size of the complex increased from 10.27 nm to 12.13 nm, and the ζ potential decreased from −6.57 mV to −16.13 mV at a drug loading ratio of 1:200, with a cumulative release of about 60% in 48 h. In a sepsis model, the system increased the survival rate of septic mice from 40% to 95% and promoted the polarization of macrophages to the anti-inflammatory M2 type, thereby effectively alleviating the systemic inflammatory response and tissue damage, and providing a new strategy for targeted therapy of neutrophil-related inflammatory diseases (Figure 3b).

**Figure 4:**
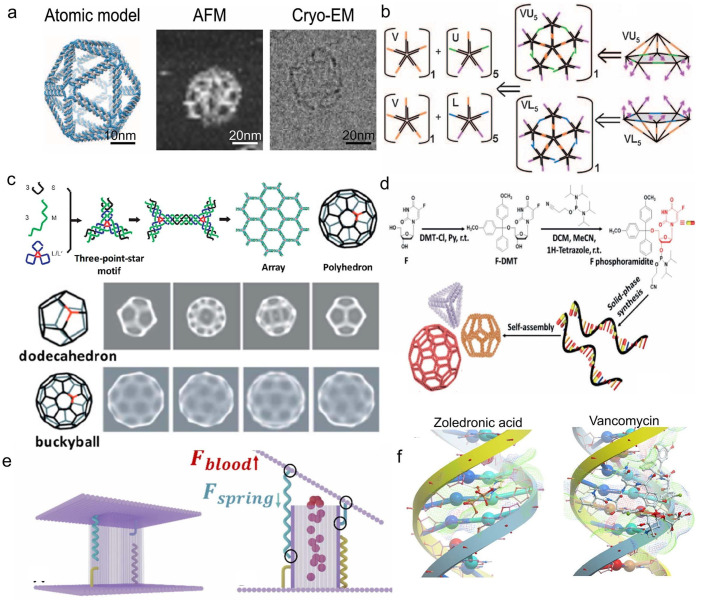
(**a**) Schematic model, AFM surface morphology images, and cryo-EM images of a DNA icosahedron. Reprinted with permission from Ref. [52]. Copyright © 2016, The American Association for the Advancement of Science. (**b**) Schematic synthetic strategy for modular assembly of a DNA icosahedron. Reprinted with permission from Ref. [53]. Copyright © 2009, WILEY-VCH. (**c**) Hierarchical self-assembly process of a DNA polyhedron, original cryo-EM images of a DNA dodecahedron and DNA buckyballs, and reconstructed DNA polyhedron from cryo-EM images. Reprinted with permission from Ref. [55]. Copyright © 2020, American Chemical Society. (**d**) Schematic synthetic strategy of the “DNA Trojan”. Reprinted with permission from Ref. [58]. © 2017, Wiley-VCH. (**e**) Schematic working principle of hemodynamic-responsive DNA origami capsules. Schematic of a shear force-responsive DNA origami capsule (DOC) for drug delivery. Elevated shear forces stretch DNA springs and open the lids to release encapsulated cargo, while the capsule remains closed under normal flow. Black circles denote attachment points. Reprinted with permission from Ref. [59]. Copyright © 2026, The Authors. (**f**) Molecular simulation images of the interaction between Z-TDN and V-TDN in drug-loaded platforms of self-assembled tetrahedral DNA nanocarriers. Reprinted with permission from Ref. [62]. Copyright © 2025, The Author(s).

In addition to tFNA, other 3D DNA polyhedral structures have also been explored by researchers, including DNA cubes [31,50], DNA dodecahedron [51], DNA icosahedron (Figure 4a,b) [52,53], and DNA fullerene spheres (Figure 4c) [54,55]. Their internal closed nanoscale cavities provide ample loading space for drug molecules, effectively protecting drug activity from degradation or inactivation in complex biological environments. Based on these structural advantages, researchers have developed various DNA polyhedral drug delivery systems for chemotherapeutic drug and therapeutic protein molecules. For example, Tam et al. [56] constructed a 3D spherical DNA nanocage for drug delivery to treat brain tumors using a modular self-assembly strategy based on the principles of supramolecular DNA self-assembly. Dox is loaded in the DNA double helix by base embedding, which significantly prolongs the circulating half-life of the nanocage in serum, and the DNA nanocage without a modified ligand shows excellent blood–brain barrier penetration, with its apparent penetration coefficient being significantly higher than that of the tubular DNA structure and ligand modification system. In the U-87 MG glioma model, the drug-loaded nanocage effectively crosses the blood–brain barrier and inhibits tumor growth, demonstrating the structural advantages of a three-dimensional DNA rigid framework in targeted drug delivery for brain tumors.

#### 2.2.2. Interaction Modes Between FNAs and Drugs

FNAs possess excellent biocompatibility, structural controllability, and spatial programmability, enabling precise drug delivery and providing a modular, customizable interaction paradigm for drug loading and delivery. The interaction modes between FNAs and drugs can be primarily categorized into covalent conjugation, spatial encapsulation, and non-covalent physical interactions.

Covalent conjugation refers to the direct attachment of drug molecules to the FNA skeleton, typically involving the phosphate backbone, nucleobases, or the termini of DNA strands, through stable or responsive covalent chemical bonds (e.g., disulfide bonds [57]). This mode allows for precise control over the drug loading site and quantity, resulting in nanomedicines with defined drug loading and uniform structure, and effectively avoiding the issues of heterogeneous loading and premature leakage associated with physical adsorption or non-specific encapsulation. To achieve a higher degree of integration between the drug and the carrier, another strategy involves incorporating chemically modified drugs into the nucleic acid backbone via solid-phase synthesis. Mou et al. [58] proposed a “DNA Trojan horse” strategy (Figure 4d), where the nucleoside analog drug floxuridine (F) was chemically modified into a phosphoramidite monomer and covalently integrated into the DNA sequence via phosphodiester bonds through solid-phase synthesis, thereby achieving structural embedding of the drug within the nucleic acid backbone. Based on this, they self-assembled tetrahedral DNA, dodecahedral DNA, and DNA buckyballs. The F-Buckyball induced apoptosis in HeLa cells at a rate of 64.6%, significantly higher than that induced by the free drug (37.2%), and achieved efficient tumor targeting and growth inhibition in vivo via the EPR effect. This demonstrates the feasibility and advantages of constructing intelligent FNA delivery systems with defined drug loading, clear structure, and programmable performance through structural integration of the drug.

Spatial encapsulation is another crucial drug loading mode for framework nucleic acids. Its core principle involves utilizing FNAs to form nanoscale containers with specific three-dimensional structures, creating geometrically defined cavities that encapsulate drug molecules through physical confinement without relying on chemical bonding between the drug and the FNA nanocontainer. This encapsulation mode not only protects drug activity but also enables precise release by leveraging the carrier’s own stimuli-responsive properties, offering excellent design flexibility. For instance, Omer et al. [59] designed a hemodynamic force-responsive DNA origami capsule (DOC) for targeted drug delivery to narrowed arteries (Figure 4e). This capsule consists of a hollow box body (32.5 × 32.5 × 50 nm^3^) and two rectangular lids (76 × 90 nm^2^) connected via DNA hinges and single-stranded DNA springs. Therapeutic molecule drugs are encapsulated within the cavity via spatial entrapment, with a theoretical loading capacity of no less than 10 molecules. Under normal blood flow (shear force ~0.1 pN), the spring remains pre-tightened, and the capsule stays closed. In narrowed arteries, where local shear force increases to 3–4 pN, the spring stretches beyond the geometric threshold of 47 nm, driving the lids to open and release the drug. This study showcases the potential of FNAs for lesion-specific drug release through mechanically responsive spatial encapsulation, offering a new strategy for precise treatment of hemodynamics-related diseases like vascular stenosis. Besides exogenous physical stimuli, spatially encapsulating systems triggered by biochemical signals (e.g., telomerase [60]) also demonstrate excellent targeting performance.

Non-covalent physical interactions involve drug molecules binding to specific sites on FNAs through weak interactions. This encapsulation mode does not rely on closed cavities or chemical bonding and typically does not require additional chemical modification of the drug molecules. The construction process is relatively straightforward, and due to its loading flexibility and ease of achieving multifunctional synergistic delivery, it is widely adopted in FNA-based drug delivery systems [61]. Building on this, utilizing the differential groove binding of the DNA double helix enables multidrug synergistic delivery. In 2025, Huang et al. [62] developed a drug loading platform based on self-assembled tetrahedral DNA nanocarriers (TDNs) for the synergistic treatment of bone defects and methicillin-resistant Staphylococcus aureus (MRSA) infections (Figure 4f). Molecular modeling results demonstrated that zoledronic acid (ZA) and vancomycin (VAN) can interact with DNA structures and may achieve co-loading through physical embedding in the DNA grooves of TDN. Drug loading in TDN significantly enhanced the overall therapeutic efficacy, with osteogenic differentiation promotion increasing by approximately threefold. Moreover, at a concentration of 0.75 minimum inhibitory concentration (MIC), the combined therapy exhibited superior antibacterial activity against MRSA compared to free VAN. This enhanced pharmacological effect may be attributed to the DNA nanostructure’s modulation of drug stability and release behavior, enabling ZA and VAN to maintain more effective local concentrations during action, thereby improving drug utilization efficiency and potentially reducing dosing requirements while mitigating resistance issues. These findings highlight the application potential of programmable nanocarriers based on DNA embedding mechanisms in combined bone repair and anti-infection therapies.

**Figure 5:**
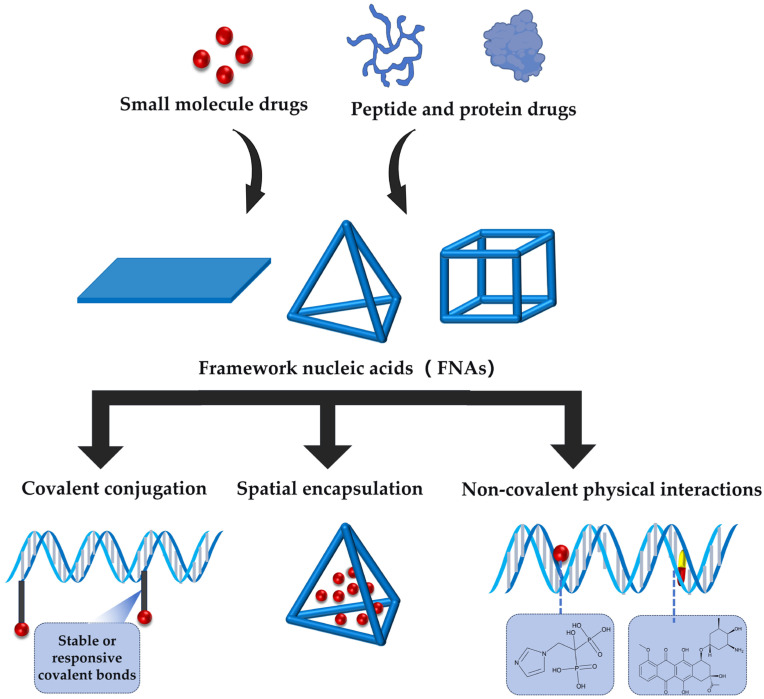
Schematic illustration of the drug loading mechanisms of FNAs for the delivery of heterogeneous molecular drugs.

In general, all three drug loading strategies using FNAs as nanocarriers provide practical routes for the delivery of heterogeneous molecular drugs (Figure 5).

## 3. Delivery of Small Molecule Drugs by FNAs

Among the different classes of marketed drugs, small molecule drugs account for approximately 80–90%, holding a clearly dominant position [63,64]. Small molecule drugs, such as anticancer agents like doxorubicin and paclitaxel and antibacterial agents like erythromycin and neomycin sulfate, have also revealed certain problems in experimental and clinical applications, including poor solubility, low bioavailability, susceptibility to drug resistance, and toxicity to normal cells due to non-specific distribution. Therefore, there is an urgent need for effective methods to overcome these difficulties, and drug delivery systems are one of the favored solutions.

As an emerging drug delivery system, FNAs possess excellent physicochemical properties that attract an increasing number of researchers to consider them a powerful tool for solving the challenges of precise targeted drug delivery. The main body of FNAs consists of highly water-soluble DNA nanostructures. When loading hydrophobic small molecule drugs, they can assist in transporting these drugs within the aqueous environment of the body to target cells. Furthermore, as a type of nanocontainer, FNAs can protect the loaded small molecules in complex physiological environments, thereby enhancing their bioavailability. Additionally, the characteristic of FNAs to be readily taken up by cells enables them to more efficiently target and deliver small molecule drugs to diseased tissues requiring treatment, potentially bypassing inherent drug resistance mechanisms [65]. The delivery of small molecule drugs by FNAs is summarized in Table 2.

### 3.1. Delivery of Small Molecule Chemotherapeutic Drugs by FNAs

As a persistent challenge in human medicine, cancer continually attracts researchers’ attention. Consequently, a vast array of anticancer drugs has been developed for cancer treatment. Chemotherapy remains an effective modality, with small molecule anticancer drugs constituting the vast majority of agents used and serving as its cornerstone. These small molecules typically include anthracycline antibiotics (doxorubicin, daunorubicin, etc.), inhibitors (camptothecin and its derivatives, paclitaxel, etc.), metal complexes (platinum complexes, ruthenium complexes, etc.), antimetabolites (gemcitabine, etc.), and photosensitizers (methylene blue, BMEPC, etc.). However, many small molecule anticancer drugs also exhibit drawbacks such as poor water solubility, low bioavailability, propensity for systemic toxicity, short in vivo half-life, and the emergence of multidrug resistance in tumor cells, which can also impact their efficacy. FNAs, with their precise geometric structures, excellent biocompatibility, programmable surface modification capabilities, and unique cell membrane permeability, have emerged as ideal nanocontainers to address the aforementioned issues. They can efficiently load small molecules via physical entrapment, chemical covalent conjugation, or non-covalent intercalation and achieve precise targeting through modification with aptamers, peptides, or antibodies.

#### 3.1.1. Delivery of Anthracycline Drugs by FNAs

Anthracycline small molecule drugs are the most widely used model drugs in FNA-based drug delivery research. Their primary anticancer mechanism involves specifically intercalating between the base pairs of the DNA double helix, thereby hindering DNA transcription and replication [66]. However, their propensity to induce drug resistance and toxicity to normal cells consistently limits their further application. Considering their inherent ability to interact with DNA and their fluorescent properties that facilitate tracking, researchers have developed methods using FNAs to deliver anthracyclines, conducting extensive work on carrier structure, targeting modifications, overcoming resistance, and intelligent responsiveness.

**Figure 6:**
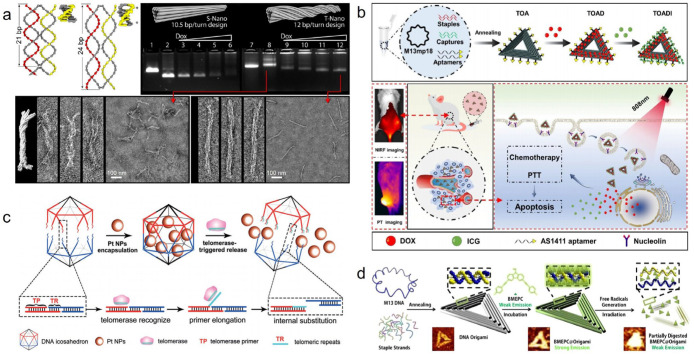
(**a**) Pitch and twist density shift after Dox intercalation (* = Dox). A 2% AGE shows differences in folding quality between the straight nanotube (S-Nano) and twisted nanotube (T-Nano) (Lane 1 and 7 = corresponding scaffold, Lane 2–6, 8–12 = gradient concentrations of Dox loaded on nanotubes from 0 to 96 μM), followed by TEM images of T-nano without Dox (left) and with 96 μM Dox (right). The arrow represents the TEM image in the corresponding lane. Reprinted with permission from Ref. [70]. Copyright © 2012, American Chemical Society. (**b**) Schematic illustration showing the co-loading of Dox and ICG on a triangular DNA origami nanostructure (TOA) with AS1411, undergoing combined chemotherapy and photothermal therapy (PTT). TOAD = TOA with Dox; TOADI = TOAD with ICG. Reprinted with permission from Ref. [74]. Copyright © 2023, The Author(s). (**c**) Progress of platinum nanoparticles (Pt NPs) folded into and released from a DNA icosahedron in the presence of telomerase. Reprinted with permission from Ref. [60]. Copyright © 2018, Wiley-VCH. (**d**) Schematic illustration shows the formation of triangular DNA origami, BMEPC loading and its function under irritation. Reprinted with permission from Ref. [115]. Copyright © 2016, American Chemical Society.

FNA nanocontainers exhibit various geometric shapes, and FNAs with different geometries can display distinct structural stability, drug loading capacity, and efficacy for the same payload molecule [67,68,69]. Zhao et al. [70] achieved highly tunable drug release rates by adjusting the structural parameters of DNA origami, such as twist and 3D conformation (Figure 6a). By designing different DNA origami configurations, the release half-life of Dox could be significantly extended from less than 10 h to over 100 h. In various breast cancer cell lines, this tunable carrier system demonstrated enhanced cytotoxicity compared to free Dox. Furthermore, another study [71], using in vivo and in vitro imaging to compare three DNA origami structures of different shapes, confirmed that DNA origami exhibits enhanced passive tumor targeting and prolonged accumulation in tumor regions, with the extent of passive tumor accumulation varying among different shapes in a breast cancer model.

Aptamer modification is a mainstream strategy to enhance DOX targeting. Aptamers are short single-stranded DNA, RNA, or peptide chains that can bind to various specific targets, thereby enhancing the targeting ability of the modified entity [72]. AS1411 is a DNA aptamer that specifically binds nucleolin, which is often overexpressed on the surface of cancer cells, making AS1411 aptamer modification an effective strategy to enhance targeting capability. Multiple studies have demonstrated that AS1411 aptamer-modified FNAs can serve as multifunctional nanoplatforms for targeted delivery of Dox or photosensitizers for combination therapy, confirming that AS1411 is the primary provider of targeting ability [73]. Li et al. [74] constructed an NIR/pH dual-triggered AS1411-modified triangular origami loaded with Dox and the photosensitizer indocyanine green (ICG) (Figure 6b), achieving combined chemo-phototherapy with a tumor inhibition rate of 90%. Wang et al. [75] developed a tetrahedron modified with AS1411 and the polyaspartic acid-based cationic polymer mPEG-PAsp (TECH) for co-delivery of Dox and the photosensitizer TMPyP4. This drug delivery system achieved a loading efficiency of up to 77% in HeLa cells. Upon near-infrared light irradiation, the reactive oxygen species (ROS) level generated was significantly higher than with single-drug treatment, and the IC50 value was substantially reduced, demonstrating the synergistic effect of combination therapy and effective lysosomal escape. Mucin 1 (MUC1) is a highly glycosylated, high-molecular-weight glycoprotein overexpressed on the surface of most adenocarcinoma cells; thus, MUC1 aptamer can also function as an effective modifying moiety for specific recognition of cancer targets [76,77,78]. Han et al. [79] constructed multivalent DNA tetrahedral nanocages (nApt-Td) modified with different numbers of MUC1 aptamers. The results showed that increasing the number of aptamers at the vertices significantly enhanced the uptake efficiency of Dox-loaded DNA nanocages by MCF-7 tumor cells, while effectively reducing non-specific uptake by normal cells. Chaithongyot et al. [80] constructed an MUC1 aptamer-functionalized DNA origami nanosphere loaded with Dox. Validation in three cell lines with different MUC1 expression levels demonstrated that this system exhibited extremely high selectivity for MUC1-overexpressing MCF-7 cells, with minimal toxicity to low-expressing cells, and the induced tumor cell apoptosis rate was significantly higher than that of equivalent concentrations of the free drug. Tyrosine-protein kinase 7 (PTK7) is a catalytically inactive receptor tyrosine kinase often highly expressed in various cancers. Sgc8 and Sgc8c are specific aptamers for PTK7. Cao et al. [81] reported a targeted delivery system based on triangular DNA origami and multivalent Sgc8 aptamers. This DNA origami structure remained structurally intact after 24 h of incubation in cell culture medium, and multivalent aptamer modification enabled more precise delivery of Dox into target cancer cells. Liu et al. [82] designed a tetrahedral DNA nanostructure loaded with Dox and modified with the sgc8c aptamer, demonstrating its ability to specifically kill PTK7-positive CCRF-CEM cells with little toxicity to PTK7-negative Ramos cells.

Beyond aptamers, proteins, antibodies, small molecule ligands, and peptides are also important targeting moieties [83,84,85,86,87]. Zhang et al. [88] prepared nanoparticles composed of a DNA tetrahedron and two Affibody molecules. Affibodies are small proteins (58 amino acids) containing a three-helix bundle domain, derived from the Z-domain scaffold of immunoglobulin G protein. Each nanoparticle could non-covalently bind approximately 53 Dox molecules. This system exhibited more than twice the inhibitory activity against HER2-overexpressing BT474 cells compared to the traditional antibody drug trastuzumab, while significantly reducing toxicity to normal cells. Zhang et al. [89] reported a method for constructing biomimetic, size-controllable, and self-degradable Sgc8 aptamer-modified cancer-targeting DNA nanoflowers (Sgc8-NFs-Fc). Experiments showed that the size of these nanostructures could be controlled by varying the amount of hydrophobic Fc-base DNA. This iron-based material-modified nanoflower could undergo self-degradation via the Fenton reaction within cancer cells, promoting drug release and thereby leading to greater nuclear accumulation of loaded Dox and enhanced cytotoxicity. Its enhanced targeting also reduced the non-specific distribution of Dox in normal organs.

Multidrug resistance (MDR) refers to the phenomenon where cells develop resistance simultaneously to multiple drugs with different structures and mechanisms of action. It is a major cause of chemotherapy failure in cancer and of difficulty in treating superbug infections. While the exact causes are not fully elucidated, it is generally believed to involve mechanisms such as increased drug efflux, decreased drug uptake, and altered drug metabolism. Using nanocomplexes for drug delivery is considered an effective method to reverse MDR, and the role of FNAs as nanoscale drug delivery containers in reversing MDR is gradually being explored by researchers [65,90,91]. Yan et al. [92] prepared a nanocomplex consisting of a disulfide-crosslinked polyethyleneimine (PSP) coating on a DNA tetrahedron (TDNs) loaded with Dox. The high positive charge density of PSP created a “pore-forming” effect on the cell membrane, allowing direct cellular entry bypassing traditional endocytosis pathways and effectively evading efflux pump recognition. This system also exhibited excellent dual responsiveness to glutathione (GSH) and DNase I, enabling rapid intracellular degradation and Dox release.

Typically, drug targeting focuses on organs, tissues, or cells; however, the actual site of drug action is often at the subcellular level, within organelles such as mitochondria. Therefore, drug delivery systems capable of direct organelle-targeted delivery are also gaining research attention. For instance, a DNA tetrahedron structure modified with a cationic amphiphilic peptide (KLA) was designed to load Dox [93]. This system achieved a Dox loading rate of approximately 77%, with tetrahedral DNA nanostructures modified with three KLA sequences (3KLA-TDNs) showing the most significant accumulation in mitochondria. This precise localization led to loss of mitochondrial membrane potential, substantial release of cytochrome c, activation of the Caspase-3/9 pathway, and increased expression levels of p21 and p53 proteins. Li et al. [94] proposed a hierarchical assembly strategy for long-chain tetrahedral DNA (Apt-Nano-Tetra) mediated by small-sized gold nanoparticles (AuNPs) acting as “nanotape”. This structure served as a nucleus-targeted drug delivery vehicle (Apt-ADMC). The Dox loading capacity was increased by 75–85 times. Systemically administered Dox-Apt-ADMC showed a sevenfold enhancement in tumor accumulation and inhibited tumor growth by nearly 100%, without detectable systemic toxicity.

Furthermore, employing drug delivery systems with intelligent response characteristics is a crucial strategy for navigating the complex in vivo environment, and the excellent programmability and addressability of FNAs pave the way for applying such features. Sun et al. [95] designed a highly integrated “guided missile-nanospacecraft” (GM-NSC) system, consisting of a gold nanoparticle core and a DNA tetrahedron corona. Each GM-NSC could accommodate 1250 Dox molecules and featured a staged, dual-targeting drug delivery process, including aptamer-mediated cancer cell internalization, intelligent intracellular separation of GM and NSC, delivery of the mitochondria-acting therapeutic agent to the final target organelle, and localized release. Gu et al. [96] innovatively constructed gold nanoparticle clusters assembled from DNA origami units with a “size-shrinking” mechanism exhibiting dual responsiveness to acidic environments. This system underwent preliminary dissociation triggered by DNA triple-helix formation at pH 6.5 and achieved complete release of loaded Dox through i-motif formation at pH 5.0. This sequential response behavior increased its penetration rate in dense tumor tissue models by threefold.

In addition to organelle targeting, FNAs equipped with protective coatings can also achieve more effective drug delivery in complex in vivo environments. Cheng et al. [97] attempted to coat the surface of DNA tetrahedra with a calcium carbonate nanoshell. This composite particle released 45% of DOX within five hours at pH 7.4; however, upon entering a pH 5.5 environment, the calcium carbonate layer dissolved, and the Dox release rate reached over 80% within five hours. Yan et al. [98] utilized nuclear localization signal peptides, tetrahedral DNA nanostructure, and disulfide-crosslinked polyethyleneimine as composite materials to load Dox, using an MMP-2 sensitive PEG hydrogel as an encapsulating material for additional loading of R837, thereby integrating these components into a complex nanodelivery platform. The combination of chemotherapy and immunotherapy employed in this strategy promoted immune stimulation, thereby effectively inhibiting tumor growth.

#### 3.1.2. Delivery of Small Molecular Inhibitor Drugs by FNAs

Inhibitor drugs typically suppress cancer cell proliferation by specifically binding to key proteins involved in tumor growth or by blocking cytoskeletal function. Common inhibitor drugs include taxanes, camptothecins, and kinase inhibitors. The poor water solubility, low bioavailability, and severe neurotoxicity associated with this class of drugs pose challenges to their clinical application. As a highly hydrophilic, biocompatible, and low-toxicity nanoscale container, FNAs serve as an excellent drug delivery system for loading these drugs to overcome the aforementioned challenges. The mainstream taxane drug is paclitaxel, which is a natural, low-toxicity, broad-spectrum anticancer agent widely used clinically, despite its very low solubility and propensity to induce drug resistance. Studies have demonstrated that drug delivery systems utilizing FNAs to load paclitaxel can significantly increase the uptake rate of poorly soluble paclitaxel in target cells and overcome drug resistance by downregulating the expression of key genes [99,100]. Addressing the challenges of crossing the blood–brain barrier and lack of specificity in glioblastoma (GBM) therapy, Shi et al. [101] constructed a dual-aptamer-modified DNA tetrahedral nanocarrier (GTG). Their study found that the uptake rate of GTG in U87MG cells was as high as 60.3%, far exceeding the 20.2% of unmodified tetrahedra. After loading paclitaxel, this complex (GPC) significantly inhibited tumor cell migration and invasion. Flow cytometry showed that the apoptosis rate induced by GPC reached 24.26%, higher than the 17.19% observed in the free paclitaxel group. Camptothecin (CPT) is also a natural small molecule with anticancer activity, but its high toxicity and extremely low water solubility limit its use. Zhang et al. [57] developed a chemical grafting method based on phosphorothioate (PS) modification, site-specifically and quantitatively conjugating carbomethyl bromide-modified CPT to PS-modified DNA strands via responsive disulfide bonds. These conjugates were subsequently self-assembled into tetrahedral DNA with a precise drug-to-carrier ratio. This design ensured drug loading while significantly improving drug solubility through the hydrophilicity of DNA. In an HCT116 tumor-bearing mouse model, the experimental group significantly inhibited tumor growth without causing the hepatorenal toxicity observed in the free CPT control group. Additionally, various other inhibitor drugs have been demonstrated to exert antitumor effects when loaded onto FNAs. Long et al. [102] designed an immunoadjuvant CpG-modified tetrahedral framework nucleic acid as a nanoscale container for delivering shikonin (SK) to treat triple-negative breast cancer (TNBC). In vivo experiments showed that the tumor inhibition rate in the CpG-DT/SK treatment group reached 74.2%. Bousmail et al. [103] constructed a spherical nucleic acid (SNA) system for delivering the anticancer drug BKM120. This system utilized the hydrophilicity and negative charge of the DNA shell to prevent the drug from crossing the blood–brain barrier and accumulating non-specifically in the brain. In vivo imaging experiments confirmed that Cy5.5-labeled nanoparticles were still highly enriched at the tumor site 24 h after intravenous injection, with extremely low fluorescence signals detected in the brain.

#### 3.1.3. Delivery of Chemotherapeutic Metal Complex Drugs by FNAs

Metal complexes are also a vital component of clinical chemotherapy. Platinum-based drugs, exemplified by cisplatin, can form intra-strand or inter-strand crosslinks with DNA bases through their hydrolysis products, thereby hindering replication and transcription. Other metal complexes, such as ruthenium complexes, can generate highly toxic reactive oxygen species at target sites to kill cancer cells. However, without a delivery carrier, the damage inflicted by these drugs on normal cells is also significant, accompanied by severe nephrotoxicity, ototoxicity, and a propensity to develop drug resistance. As highly biocompatible nanoscale containers, FNAs can load metal complex drugs, delivering them specifically to cancer cells, thereby reducing toxicity to normal cells and potentially overcoming drug resistance. Furthermore, it has been reported that metal complexes can also serve as stabilizers for FNA delivery systems, effectively slowing down their degradation in physiological environments [104].

Platinum-based drugs are the mainstream metal complex anticancer drugs, with cisplatin being the first developed platinum compound exhibiting anticancer effects. Zhang et al. [105] constructed a cisplatin-loaded DNA tetrahedral nanoparticle conjugated with a HER2 affibody. This cisplatin-loaded nanoparticle demonstrated high selectivity and inhibitory effects against HER2-overexpressing breast cancer BT474 cells, while showing lower toxicity to HER2-low-expressing MCF-7 cells. At the same cisplatin concentration of 33.3 μM, the growth inhibition rate of this nanomedicine against BT474 cells was 11.67% higher than that of cisplatin alone. Furthermore, various other platinum-based drugs, such as phenanthriplatin [106], 56MESS [107], and platinum (IV) prodrugs [108], have been shown to enhance cancer cell targeting, increase anticancer activity, and reduce systemic toxicity when loaded onto FNAs. Ma et al. [60] utilized icosahedral DNA as nanocontainers to load platinum nanoparticle drugs, successfully achieving a telomerase-responsive release mechanism for targeted anticancer therapy (Figure 6c). To determine the maximum load capacity of the system, they synthesized PtNPs with different diameters (2, 4, and 10 nm) and encapsulated them within DNA icosahedrons. Compared to 10 nm PtNPs, DNA icosahedrons could encapsulate a larger number of 2 nm and 4 nm species. The 2 nm PtNPs were ultimately selected due to their large specific surface area, which facilitates rapid release of platinum ions to kill cancer cells. The response mechanism involves telomerase primers and telomeric repeat sequences integrated into the icosahedral FNAs, which disintegrate the nanocapsules through chain substitution reactions under the telomerase environment of cancer cells, thereby releasing the encapsulated platinum nanoparticle drugs and significantly inhibiting tumor growth.

Apart from platinum drugs, Huang et al. [109] developed a biotin-modified triangular DNA origami structure for delivering a ruthenium polypyridyl complex (RuPOP). Unlike free RuPOP or FNAs alone, this drug delivery system translocated to the nucleus after cellular uptake and underwent self-cleavage in the presence of DNase, triggering drug release and inducing ROS-mediated apoptosis. The study also demonstrated significant differences in the uptake levels of this system between cancer cells and normal cells, a characteristic that could also reduce systemic toxicity.

#### 3.1.4. Delivery of Antimetabolite Drugs by FNAs

Antimetabolite drugs are a class of compounds structurally similar to normal cellular metabolites. They interfere with normal biochemical reactions by specifically binding to metabolic enzymes, thereby antagonizing metabolic functions. For instance, gemcitabine is a standard treatment option for various cancers, particularly for pancreatic cancer and non-small-cell lung cancer (NSCLC). However, the clinical application of this class of drugs faces numerous challenges, including systemic toxicity due to non-specific distribution and the propensity of cancer cells to develop resistance mechanisms against antimetabolites. FNAs can act as nanocontainers for these drugs, reducing systemic toxicity through cancer cell targeting and overcoming resistance by circumventing resistance mechanisms [110]. Li et al. [111] conjugated cetuximab with tetrahedral DNA framework via click chemistry to prepare antibody–DNA nanostructure conjugates (ADNCs). Gemcitabine was integrated into the framework nucleic acid structure during the DNA tetrahedron assembly process to form a complete drug delivery system. This allowed FNAs to function effectively as loading vehicles. The results demonstrated that this system induced cancer cell apoptosis through the same mechanism as free gemcitabine, without observable significant cytotoxicity.

#### 3.1.5. Delivery of Photosensitizer Drugs by FNAs

Photodynamic therapy (PDT) is also a highly regarded cancer treatment strategy. Compared to chemotherapy, which may cause severe adverse reactions and systemic toxicity, PDT offers specific spatiotemporal selectivity and minimal invasiveness and has been widely used in clinical cancer treatment. Photosensitizers are the core components of photodynamic therapy. The primary mechanism of action of photosensitizers in PDT involves the absorption of light at a specific wavelength, exciting the molecule from the ground-state to a singlet state, followed by intersystem crossing to a long-lived triplet state. The triplet state photosensitizer can then transfer energy to surrounding ground-state oxygen molecules, generating highly cytotoxic reactive oxygen species (ROS). These ROS rapidly oxidize and damage nearby cell membranes, mitochondria, and DNA, directly killing tumor cells. However, most photosensitizer molecules exhibit poor solubility in physiological environments and low quantum yields, which directly impact their anticancer efficacy. As hydrophilic nanocontainers, FNAs have emerged as reliable carriers for photosensitizers, overcoming their hydrophobicity and enabling targeted delivery to cancer cells to exert ROS-mediated pro-apoptotic effects [74,112,113,114]. Zhuang et al. [115] employed a strategy of loading the carbazole derivative 3,6-bis[2-(1-methylpyridinium) ethynyl]-9-pentylcarbazole diiodide (BMEPC) onto triangular DNA origami (Figure 6d). This photosensitizer carries a positive charge, facilitating more effective binding to the negatively charged DNA double helix. This loading strategy effectively ameliorated the poor water solubility of BMEPC, and the binding of BMEPC to the DNA origami nanocontainer effectively reduced its photobleaching. Furthermore, the restriction of intramolecular rotation enhanced its fluorescence emission and free radical generation, thereby effectively boosting its therapeutic efficacy against cancer cells. Yan et al. [116] utilized AS1411-modified targeting tetrahedral DNA nanostructures and the immunoadjuvant CpG to construct a multidrug co-loading system (ACT@DM) carrying methylene blue and Dox for multimodal combined chemo-phototherapy-immunotherapy. Studies showed that ACT@DM could effectively generate ROS and promote cellular uptake through photochemical internalization. Moreover, light irradiation induced an immunogenic cell death (ICD) effect with this system, activating immune cells, promoting dendritic cell maturation and secretion of related cytokines, and leading to T lymphocyte activation and infiltration. To investigate the impact of chemotherapy on cancer cell hypoxia, Zeng et al. [117] used the reduced form (AQ4) of banoxantrone (AQ4N) as a model chemotherapeutic drug and photoacoustic imaging probe, loading it onto triangular DNA origami FNAs as a nanoscale container. This composite drug served as a theranostic nanoplatform integrating photoacoustic imaging (PAI) monitoring and cancer therapy. By comparing photoacoustic imaging results with the distribution of deoxy-/oxy-hemoglobin, they successfully observed an increase in oxygen saturation within cancer cells during chemotherapy. This finding also partially explains why the efficacy of prodrugs designed to be activated in situ by the hypoxic tumor microenvironment to release anticancer active molecules may not always meet expectations.

### 3.2. Delivery of Drugs for Other Diseases by FNAs

FNA nanocontainers have also been utilized to load a variety of small molecule drugs, demonstrating immense potential in the treatment of diseases in orthopedics [118,119,120,121,122,123], dermatology [124,125,126], stomatology [127], dentistry [128], ophthalmology [129], otorhinolaryngology [130], as well as in anti-infection [131,132,133,134,135] and organ protection [136] applications. The favorable tissue permeability, anti-inflammatory and antioxidant properties, excellent targeting capability, low toxicity, and high solubility of FNAs are the primary factors contributing to their appeal in these fields. Deng et al. [137] employed tetrahedral framework nucleic acids as carriers to load L-ascorbic acid 2-phosphate (AA2P), synthesizing a novel nanoplatform capable of restoring corneal transparency and function. The study demonstrated that this system significantly inhibited oxidative stress, mitochondrial membrane potential abnormalities, and apoptosis in corneal epithelial progenitor cells (TKE2) under alkaline conditions. It also regulated the expression of key markers such as K14, P63, and SOX2, and successfully exhibited excellent therapeutic effects in a mouse model of corneal alkali burn (CAB). Liu et al. [138] utilized tetrahedral framework nucleic acids as carriers to deliver a small molecule inhibitor (smI) specifically targeting glucosyltransferase C (GtfC) activity. This small molecule inhibitor suppressed GtfC activity and exopolymer formation in Streptococcus mutans without affecting bacterial viability. This system was experimentally confirmed to reduce the severity of dental caries while maintaining oral microbial diversity, exhibiting good local and systemic biosafety. In another study, Liu et al. [139] employed tetrahedral framework nucleic acids as delivery vehicles for quercetin (Que). Leveraging the inherent physiological properties of tetrahedral framework nucleic acids themselves, such as their ability to aid in anti-inflammation and antioxidation, this system was used to alleviate lipopolysaccharide (LPS)-induced sepsis. This composite structure (tFNA-Que) offered advantages including simple synthesis, stable performance, good biocompatibility, excellent water solubility, and superior anti-inflammatory and antioxidant properties. It effectively enhanced the anti-inflammatory effects of Que and tFNA by modulating the ERK/NF-κB pathway while also effectively boosting their antioxidant capacity by regulating the Nrf2/HO-1 pathway.

**Table 2:** Representative species of small molecule drugs delivered by FNAs.

Drug Type	Representative Species	FNA Framework Optimization Strategy	Mechanism of FNA	Refs.
Small Molecule Chemotherapeutic Drugs	Anthracycline Drugs	Shape optimization	Structural stabilization, increase in capacity and efficacy	[67,68,69,70,71]
		Functionalized modification	Targeted delivery and release, MDR reversal	[72,73,74,75,76,77,78,79,80,81,82,83,84,85,86,87,88,89,90,91,92,93,94,95,96,97,98]
	Inhibitor Drugs	Functionalized modification	Increase in cellular uptake, reduction in systemic toxicity	[99,100,101,102,103]
	Metal Complex Drugs	Shape optimization	Targeted delivery, reduction in systemic toxicity, structural stabilization	[104,105,106,107,108,109]
	Antimetabolite Drugs	Functionalized modification	Reduction in systemic toxicity, overcoming resistance	[110,111]
	Photosensitizer Drugs	Functionalized modification	Targeted delivery, overcoming hydrophobicity	[112,113,114,115,116,117]
Small Molecule Drugs for Other Diseases	L-ascorbic acid 2-phosphate (AA2P), quercetin (Que) et al.	Complementary DNA sequence self-assembly	Targeted delivery, overcoming hydrophobicity	[137,139]

## 4. Delivery of Peptide and Protein Drugs by FNAs

Peptide drugs and protein drugs, as core components of the biopharmaceutical field, demonstrate an irreplaceable role in modern clinical treatment. Peptide drugs are typically composed of fewer than 50 amino acids and include antimicrobial peptides, pro-apoptotic peptides, and homing peptides. Protein drugs, with larger molecular weights and more complex structures, encompass monoclonal antibodies, functional enzymes, cytokines, and growth factors. These biological macromolecules offer advantages such as high biological activity, strong specificity, and low toxicity, making them widely applicable in areas like targeted cancer therapy, metabolic disease regulation, immune modulation, and tissue regeneration and repair. However, their widespread application in in vivo experiments and clinical settings also faces numerous challenges. Due to their chemical nature, peptide and protein drugs are often unstable in physiological environments and are susceptible to rapid degradation by proteases, leading to short circulation half-lives. Concurrently, the relatively large molecular weight and charge of proteins and peptides hinder their effective penetration of hydrophobic cell membrane barriers, limiting their application to intracellular targets. Furthermore, poor targeting can lead to non-specific drug distribution, which not only reduces the local effective concentration but may also trigger significant systemic immunogenicity or toxic side effects [140].

FNAs, as precise nanocontainers constructed through DNA self-assembly technology, offer an exceptional solution to overcome these challenges. FNAs possess precisely programmable three-dimensional spatial configurations, enabling the protection of peptides or proteins by encapsulating them within internal cavities, significantly enhancing their stability. Moreover, the excellent biocompatibility and cellular internalization mechanisms of FNAs can facilitate the penetration of cell membranes by the entire delivery system, greatly improving cellular uptake efficiency. The high addressability of FNAs also makes targeted modification a feasible strategy, thereby enabling specific drug enrichment at lesion sites and controlled release [141]. The delivery of peptide and protein drugs by FNAs is summarized in Table 3.

### 4.1. Delivery of Peptide Drugs by FNAs

Peptide drugs, including antimicrobial peptides, antitumor peptides, and pro-healing peptides, show great potential as biological macromolecular therapeutics in disease treatment. However, their clinical application is limited by issues such as poor stability, susceptibility to enzymatic degradation, low cellular uptake efficiency, and lack of targeting. In recent years, FNAs, as programmable nanocontainers, have been increasingly developed as efficient delivery vehicles for peptide drugs due to their excellent addressability, programmability, and high intracellular delivery efficiency [142].

**Figure 7:**
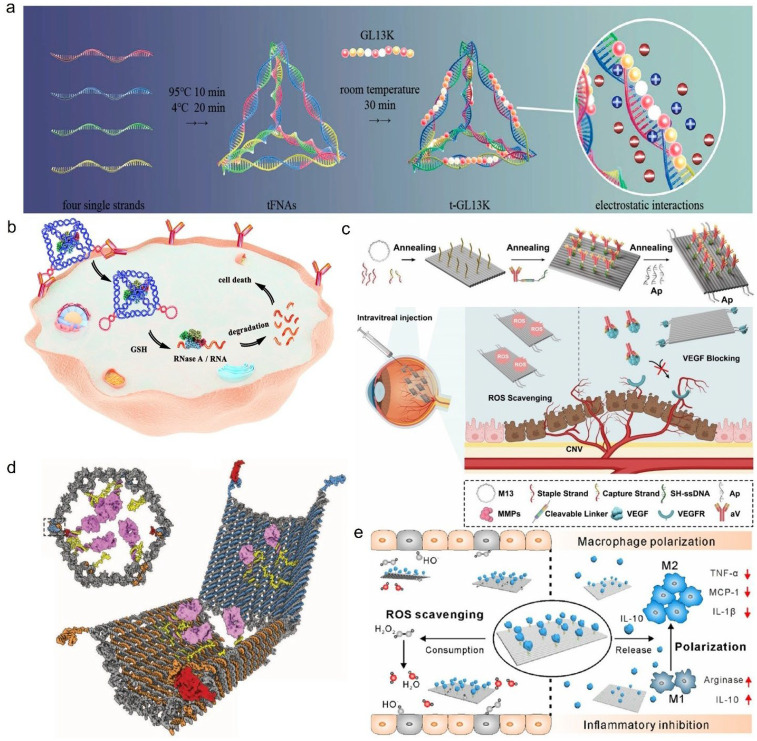
(**a**) Schematic diagram of tFNAs/GL13K complex synthesis. Reprinted with permission from Ref. [143]. Copyright © 2020, American Chemical Society. (**b**) Intracellular delivery of asdTDF−/RNase A with targeting and glutathione-induced reversible chemical bond cleavage triggering apoptosis. Reprinted with permission from Ref. [147]. Copyright © 2020, American Chemical Society. (**c**) Schematic diagram of a multifunctional DNA origami platform for targeted and combined treatment of choroidal neovascularization. Reprinted with permission from Ref. [149]. Copyright © 2024, American Chemical Society. (**d**) Schematic diagram of DNA nanorobot design. Reprinted with permission from Ref. [150]. Copyright © 2012, The American Association for the Advancement of Science. (**e**) Schematic diagram of IL-10@rDON anti-inflammatory and reactive oxygen species scavenging activity. The arrows indicate the changes in inflammatory and polarization markers: the levels of TNF-α, MCP-1 and IL-1β decrease, suggesting a weakened pro-inflammatory (M1) response; while the levels of IL-10 and arginase increase, which are consistent with enhanced anti-inflammatory activity and the M2 polarization state. Reprinted with permission from Ref. [151]. Copyright © 2024, American Chemical Society.

Antimicrobial peptides primarily function by physically disrupting bacterial cell membranes or modulating the host immune system. GL13K is a positively charged antimicrobial peptide that can kill susceptible bacteria like Escherichia coli, but it is easily degraded by proteases from Porphyromonas gingivalis. Liu et al. [143] first utilized electrostatic interactions to conjugate tetrahedral framework nucleic acids (tFNAs) with the GL13K peptide, forming a tFNAs/GL13K complex (t-GL13K) (Figure 7a). In this complex, tFNAs acted as a nanocontainer, not only increasing the local concentration of GL13K but also shielding the peptide from protease degradation like a protective shield. Experimental results showed that the antibacterial effect of t-GL13K against E. coli increased from 85% (free GL13K) to 99%. Furthermore, against P. gingivalis, which originally could degrade GL13K, the bacteriostatic rate using this delivery system increased from 1% to 32%. Additionally, stability tests demonstrated that t-GL13K was more stable in serum compared to free GL13K. His-5, an important natural antimicrobial peptide effective against Candida albicans, faces clinical challenges of a short half-life and low efficiency in penetrating the fungal cell wall. Zhang et al. [144] utilized tFNAs as a carrier for His-5, constructing a TDN/His-5 complex. The study found that tFNAs significantly enhanced the cellular internalization efficiency of His-5, allowing it to accumulate more effectively inside fungal cells, thereby significantly enhancing its killing effect and inhibiting hyphae formation. Furthermore, the complex effectively shielded His-5 from degradation by proteases in serum, greatly prolonging its duration of action. In an immunosuppressed mouse model, this FNA–drug complex not only exhibited excellent antifungal activity but also accelerated collagen deposition and bone mineralization at bone defect sites by improving the local microenvironment.

Antitumor peptides curb tumor proliferation by inducing tumor cell apoptosis, inhibiting angiogenesis, or remodeling the immune microenvironment. The KLA peptide (KLAKLAKKLAKLAK) is an antitumor peptide that targets mitochondria. It kills tumor cells by disrupting the mitochondrial membrane potential. Its main challenges are poor cell membrane penetration and susceptibility to degradation. To address this, Liu et al. [145] designed a novel approach. They synthesized a fusion protein (PMK) and linked it via a disulfide bond to a dendritic DNA framework modified with a targeting aptamer (XQ-2d), ultimately constructing a protein–DNA nanostructure. Upon reaching the target (tumor microenvironment), this structure is activated by matrix metalloproteinase 2 (MMP2), releasing the KLA peptide. The released KLA reduced ATP levels within tumor cells, thereby reversing multidrug resistance. The results indicated that, in a drug-resistant breast cancer model, this structure significantly inhibited tumor growth and reduced systemic toxicity.

Pro-healing peptides can mimic the function of vascular endothelial growth factor (VEGF) by binding to relevant receptors, activating the proliferation, migration, and differentiation of endothelial cells (ECs), thereby promoting angiogenesis and restoring blood supply in tissues. Wang et al. [146] designed a type of triangular DNA origami nanostructure (tDON) system loaded with the QKCMP peptide. By precisely arranging peptide capture strands on tDONs, they achieved efficient peptide loading and controlled release in the target environment. Atomic force microscopy (AFM) measurements showed that tDONs were approximately 10 nm in height and 120 nm in outer edge length. Biological experimental results demonstrated that this drug delivery system exhibited excellent pro-angiogenic and vascular repair capabilities in both zebrafish and mouse models.

### 4.2. Delivery of Protein Drugs by FNAs

Protein drugs, including antibodies, enzymes, and cytokines, typically exert their therapeutic effects through biocatalysis, highly specific antigen binding, or complex intercellular signaling. With the advancement of biotechnology, their potential as therapeutic agents in cancer treatment is significant. Compared to traditional chemical drugs, protein drugs offer advantages like high biocompatibility and regulatable biological functions. However, the in vivo delivery of these drugs faces challenges such as susceptibility to enzymatic degradation, poor cell membrane permeability, and short in vivo half-lives. FNAs, through their highly programmable nature, enable precise positioning of proteins inside or on the surface of the carrier. By leveraging the local high-concentration environment and spatial shielding provided by the DNA nanostructure, enzyme catalytic activity can be effectively maintained, and local controlled release of antibodies and cytokines can be achieved through targeted modifications, thereby reducing side effects.

Enzymes, as catalytic proteins, are primarily used in cancer therapy for prodrug activation and metabolic regulation. However, enzymes are prone to inactivation in vivo and are difficult to deliver effectively to target cells. FNAs, with their good biocompatibility and programmability, can provide protection for enzymes and enable precise delivery to targets, offering an ideal platform for enzyme delivery. Ribonuclease A (RNase A) degrades RNA, thereby preventing tumor cells from synthesizing proteins and ultimately inducing apoptosis. Li et al. [147] developed a fully enclosed DNA tetrahedral structure to encapsulate RNase A. In this study, RNase A was stoichiometrically encapsulated within the tetrahedral cavity via reversible chemical bonds (Figure 7b). Furthermore, bivalent aptamer sequences modified on the surface of the tetrahedral FNAs enabled precise delivery to cancer cells. Subsequent cleavage of the reversible bonds by endogenous glutathione within cancer cells triggered the traceless release of the native therapeutic protein from the DNA framework, effectively inducing apoptosis in specific cancer cells. The cascade reaction system involving glucose oxidase (GOx) and horseradish peroxidase (HRP) can be used for synergistic therapy. Kong et al. [148] constructed an FNA-confined enzyme cascade (FNA-CEC) system, precisely assembling GOx and HRP on a DNA tetrahedron. GOx catalyzed glucose consumption for starvation therapy, while the produced hydrogen peroxide was utilized by HRP to activate the prodrug indole-3-acetic acid (IAA), generating free radicals to kill tumor cells. Due to the nanoscale spatial confinement of the enzymes, the transport efficiency of the intermediate product (hydrogen peroxide) was significantly enhanced, thereby improving the therapeutic effect.

Antibody drugs are widely used in targeted therapy due to their high specificity and affinity. However, intracellular delivery of antibodies remains challenging. FNAs can achieve specific antibody delivery by modifying targeting units. Wu et al. [149] developed a rectangular DNA origami platform (aV-cL-Ap-rDOS) loaded with anti-VEGF antibody (aV) and a VEGF aptamer (Ap) (Figure 7c), connected via a matrix metalloproteinase (MMP)-cleavable peptide. This platform utilized the dual-targeting ability of aV and Ap to enrich at choroidal neovascularization (CNV) lesions, subsequently releasing the antibody upon MMP cleavage. The Ap synergistically blocked VEGF, while the DNA origami itself scavenged reactive oxygen species (ROS), reducing oxidative stress. Experiments showed a significant reduction in neovascular area in a laser-induced CNV mouse model treated with aV-cL-Ap-rDOS, with good ocular safety and no observed intraocular pressure abnormalities or retinal toxicity. Douglas et al. [150] designed an autonomous nanorobot composed of DNA origami, shaped like a 35 × 35 nm hexagonal barrel and controlled by aptamer-based gates (Figure 7d). This robot could recognize different cell types (e.g., Kasumi-1 vs. Ramos cells) and decide whether to open and display its internal molecular payload based on logic gate operations (AND/OR). In experiments, combinations of anti-human CD33 antibody and anti-human CDw328 antibody Fab’ fragments were loaded onto the robot, successfully inhibiting Jun N-terminal kinase (JNK) and Akt (protein kinase B) signaling upon payload presentation.

Cytokines, such as interleukins (ILs), play roles in regulating immune responses and promoting tissue repair, but their short in vivo half-lives and tendency to cause systemic side effects limit their use. DNA nanocarriers enable local controlled release of cytokines. Fan et al. [151] constructed a rectangular DNA origami nanostructure loaded with IL-10 (IL-10@rDON) for treating acute kidney injury (AKI) (Figure 7e). Through the synergistic anti-inflammatory and antioxidant effects of IL-10@rDON, renal function was significantly improved, and tissue regeneration was promoted. Mice treated with IL-10@rDON showed significantly reduced levels of blood urea nitrogen (BUN), uric acid, and serum creatinine (Crea), and pathological sections indicated a marked decrease in renal tubule injury scores.

**Table 3:** Representative FNA-based delivery systems for peptides and proteins.

Drug Type	Representative System	Drug Loading Strategy	Mechanism of FNA	Main Biological Effects	Ref.
Peptides	tFNA/GL13K	Electrostatic attraction	Spatial protection, local enrichment	Improved stability and antibacterial efficacy	[143]
	TDN/His-5	Electrostatic attraction	Cellular internalization enhancement, enzymatic degradation resistance	Hyphal formation inhibition	[144]
	PGD (KLA)	Covalent bonding	Targeted delivery, microenvironment-responsive release	Tumor growth suppression and multidrug resistance reversal	[145]
	tDONs/QKCMP	Site-specific capture strands	Targeted delivery and release	Pro-angiogenic capacity promotion	[146]
Proteins	TDF/RNase A	Reversible covalent bonding	Spatial protection, targeted delivery, GSH-responsive release	Precise cancer cell apoptosis	[147]
	FNA-CEC	Covalent bonding	Synergistic effect of spatial confinement and cascade reaction	Catalytic efficiency improvement	[148]
	aV-cL-Ap-rDOS	MMP-cleavable peptide linker	Targeted accumulation, enzyme-responsive release	Oxidative stress and neovascularization reduction	[149]
	DNA robot	Complementary DNA sequence self-assembly	Targeted delivery, cargo exposure	Specific signal regulation	[150]
	IL-10@rDON	Site-specific capture	Targeted accumulation, prolonged retention; ROS scavenging	Reduction in levels of specific molecules and enhancement of anti-inflammatory effects	[151]

## 5. Biological Effects and Pharmacodynamics of Framework Nucleic Acid Composite Drugs

As an emerging nanoscale drug delivery platform, the ultimate application value of FNA composite drugs depends on a series of biological effects and pharmacodynamic behaviors exhibited within complex biological systems. These behaviors span multiple scales, from the molecular and cellular levels to living organs and tissues, constituting a complete dynamic process from cellular entry to the exertion of therapeutic effects and eventual clearance from the body [152]. A deep understanding and proactive regulation of this process form the core scientific basis for their clinical translation (Table 4).

### 5.1. Cellular-Level Effects

**Figure 8:**
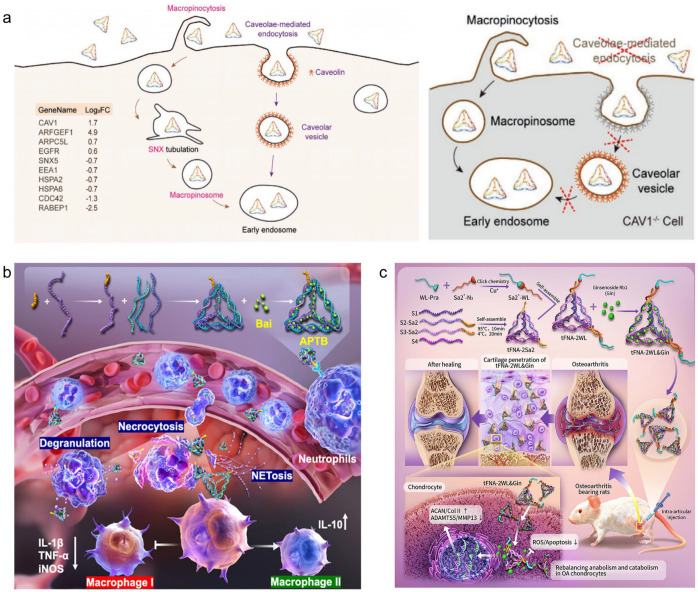
(**a**) Schematic illustration showing two routes of endocytosis of tetrahedral DNA nanostructures (TDN), along with the levels of ten differentially expressed proteins and the inhibition of one caveolae-related route by knocking out of CAV1 in cells. The cross symbol (x) symbol represents the artificial blocking or inhibition of the specific intracellular endocytic pathway. Reprinted with permission from Ref. [155]. Copyright © 2021, Wiley-VCH. (**b**) Schematic diagram showing the formation of DNA tetrahedron and APTB loading of Bai, as well as the processes of recognition, necrocytosis and degranulation in vivo. Reprinted with permission from Ref. [46]. Copyright © 2025, American Chemical Society. (**c**) Schematic diagram shows the design of tFNA−2WL and tFNA−2WL & Gin, followed by the intra-articular injection in rats, uptake by chondrocytes, and the mechanism of Gin release and cartilage protection against osteoarthritis. The fluorescence intensities of Col II and ACAN (anabolic factors) as well as MMP13 and ADAMTS5 (catabolic factors) reflect the metabolic balance of chondrocytes. tFNA&Gin and tFNA−2WL&Gin inhibited the upregulation of MMP13 and ADAMTS5, while enhancing the expression of Col II and ACAN, indicating that the metabolic balance of the extracellular matrix has been restored. Reprinted with permission from Ref. [44]. Copyright © 2025, The Author(s).

The cellular uptake mechanism of FNA composite drugs is not a simple ligand–receptor binding process but a complex kinetic phenomenon co-regulated by ligand type, size, affinity, spatial arrangement, and receptor density [153]. FNAs exhibit efficient cellular uptake mechanisms, primarily relying on energy-mediated endocytosis. Zhu et al. [154] treated keratinocytes (HaCaT cell line) and fibroblasts (HSF cell line) with Cy5-tFNA or Cy5-single strand DNA (ssDNAs) to investigate their uptake capacity for tetrahedral framework nucleic acids (tFNAs). Results showed that after 10 h of treatment, the Cy5 fluorescence intensity in cells exposed to tFNAs was significantly higher than that in the Cy5-ssDNAs group, indicating that tFNAs were readily taken up by both cell lines and that the cellular uptake efficiency of tFNAs was higher than that of ssDNAs. To clarify the cellular uptake mechanism of framework nucleic acids and overcome the interference of traditional fluorescent tracer labeling, Tian et al. [155] proposed a label-free proteomic integration strategy to systematically screen key target proteins related to tetrahedral DNA nanostructure (TDN) endocytosis (Figure 8a). Using microscale thermophoresis (MST) to verify binding affinity, CRISPR/Cas9 gene knockout experiments, and pathway-specific inhibitor assays, they demonstrated the mechanism by which framework nucleic acids achieve efficient cellular uptake through the synergistic action of two pathways: caveolin (CAV1)-mediated endocytosis and macropinocytosis. Furthermore, besides investigating uptake mechanisms, studying cellular uptake efficiency is equally important. Bai et al. [119] showed that the cellular uptake rate of quercetin alone was only 2.5%, but when complexed with tFNAs, the uptake rate reached 12.9%, representing a fivefold increase. Similarly, Zhou et al. [46] connected the targeting peptide Ac-PGP to tFNAs vertices via click chemistry and then loaded baicalin through hydrophobic interactions to form APTB nanocomplexes. Fluorescence staining results showed that after six hours of treatment, minimal amounts of baicalin alone or tFNAs alone were taken up by neutrophils, whereas a large amount of material from the APTB group was absorbed by neutrophils (Figure 8b). These findings indicate that the cellular uptake rate of composite drugs is significantly higher than that of the drugs alone.

Notably, in studies using FNAs as drug nanocontainers, enhancing their cellular uptake efficiency is a key step in improving delivery efficacy. Besides their inherent endocytic properties, specific chemical modifications can significantly enhance their ability to enter cells and their specificity. Li et al. [156] modified the surface of siRNA-templated spherical framework nucleic acids (ST-SFNAs) with catalase, enabling them to catalyze the production of oxygen bubbles from high concentrations of H_2_O_2_ in tumors, thereby providing chemotactic power to drive the active migration and enrichment of the carrier towards the tumor. Simultaneously, modifying the outer lipid membrane with folic acid (FA) allowed for high-specificity binding to folate receptors (FRs) overexpressed on tumor cell surfaces, efficiently initiating receptor-mediated endocytosis. Results showed that this dual-modification strategy significantly enhanced cellular uptake and penetration depth into 3D tumor spheroids (reaching 130 μm).

After entering cells, the primary challenge for FNA composite drugs is endosomal/lysosomal escape, i.e., avoiding capture and degradation within lysosomes during intracellular transport to ensure that the drug can reach its intracellular target and exert its efficacy [157]. Hu et al. [158] constructed a dendritic FNA polymer (FNA dendrimer) composed of five tetrahedral DNA monomers and used single-particle tracking technology to observe the entire process of its endocytosis, inter-organelle transport, and exocytosis in real time within living cells. The study found that this structure was efficiently internalized via the caveolin pathway. Following internalization, vesicles carrying the FNA dendrimer primarily underwent rapid, directed long-distance transport along the microtubule network, entering the endo-lysosomal pathway and eventually being slowly exocytosed. Therefore, investigating the delivery mechanisms of FNA composite drugs is crucial. Pal et al. [85] utilized DNA origami nanostructures as carriers, modified with folate for targeting, and successfully loaded the anticancer drug Dox for treating folate receptor 1 (FOLR1)-overexpressing triple-negative breast cancer (TNBC). Confocal fluorescence microscopy and co-localization experiments revealed that after cellular entry, the DNA origami nanostructures were primarily internalized via endocytosis into endosomes and eventually transported to acidic lysosomes. Furthermore, experiments observed that while the DNA origami carrier itself remained in lysosomes, the loaded Dox could be released from the carrier and escape the lysosomal/endosomal compartments. In FOLR1-overexpressing MDA-MB-468 cells, the IC50 value of Dox delivered by folate-origami was approximately 31-fold and 10.2-fold lower than that of free Dox and non-targeted origami, respectively. This achieved not only targeted intracellular drug delivery but also enhanced drug efficacy.

**Table 4:** Biological effects and pharmacodynamics of FNA composite drugs.

Biological Stage/Level	Key Factor/Mechanism	Impact on Biological Behavior	Refs.
Cellular-Level Effects	Cellular Internalization	Energy-mediated endocytosis, increase in uptake efficiency	[46,119,154,155]
	Receptor–Ligand Interaction	Targeted and specific recognition	[156]
	Intracellular Transport and Escape	Endosomal or lysosomal escape, avoidance of degradation	[157,158]
In Vivo Kinetic Behavior	Blood Circulation and Clearance	Rapid clearance	[159]
	Biodistribution, Lesion Site Enrichment and Metabolism	Specific accumulation, regulation of metabolic rate	[160,161]
	Safety and Biocompatibility	Low organ toxicity, high uptake rate	[44,61,152]

### 5.2. In Vivo Efficacy and Distribution

In living organisms, the therapeutic efficacy of FNA composite drugs depends on their residence time in blood circulation, their ability to accumulate at the lesion site, and their eventual clearance pathway—these three factors constitute the core triangle of their in vivo pharmacodynamics. Wamhoff et al. [159] systematically elucidated the in vivo kinetic behavior of unmodified wireframe DNA origami nanoparticles in mice following intravenous administration. The study found that nanoparticles were rapidly cleared from the blood circulation post-injection, largely leaving the systemic circulation within one-hour. In vivo imaging showed that one-hour post-injection, fluorescence signals peaked in the liver, indicating the liver as the primary organ for nanoparticle accumulation. However, liver signals decreased significantly after four hours, accompanied by increased fluorescence in the bladder region, suggesting that the particles were degraded by nucleases in vivo into small fragments and ultimately eliminated via renal filtration.

To further confirm the generality of FNA in vivo behavior and establish quantitative relationships between structural parameters (such as size) and pharmacokinetics, subsequent studies employed comparative designs. Guo et al. [160] evaluated the pharmacokinetics, immunogenicity, and immunotoxicity of pure tetrahedral DNA nanostructure (TDN) and DNA–polymer nanoparticles (DNPs) in animal models. Their study showed that the half-life (t_1_/_2_ ≈ 9.88 min) of small-sized TDNs was significantly shorter than that of larger DNPs (t_1_/_2_ ≈ 19.80 min), intuitively demonstrating that nanoparticle size is a key parameter regulating in vivo circulation time. Concurrently, in vivo imaging revealed that both types of particles predominantly accumulated in the liver and kidneys within one half-life post-injection, validating the generality of the liver–kidney enrichment and rapid clearance pattern. However, this prevalent clearance pathway, often viewed as a biological barrier, precisely provides a starting point and target for rational design. Research indicates that by precisely modulating the size, shape, surface chemistry, and administration route of framework nucleic acids, the in vivo fate can be reshaped from passive systemic clearance to active organ targeting [161].

Furthermore, investigating the enrichment of FNAs as nanoscale containers at tumor/lesion sites is also highly important. Wiraja et al. [61] selected Dox as a model drug, loaded it via intercalation into an optimal 21-base-pair framework nucleic acid (TH21), and conducted in vivo efficacy and safety validation experiments. In a mouse subcutaneous melanoma model, topical application of a cream containing TH21-loaded Dox (TH-Dox) resulted in drug accumulation at the tumor site that was 3 times (at 24 h) to 5.67 times (in deeper tumor regions) higher than that of free Dox. This indicated that TH-Dox could deliver Dox to tumor tissue at depths of 400–450 μm subcutaneously, whereas free Dox only remained in the superficial skin (approximately 50–75 μm). Meanwhile, TH-Dox treatment reduced tumor volume to 33% of the control group, demonstrating significantly superior tumor suppression compared to free Dox and other nanocarriers. This highlights that FNAs achieved efficient local accumulation and therapeutic effects while minimizing systemic toxicity and administration-related damage. Currently, relevant literature has reported enrichment data for framework nucleic acids in over a dozen organs/tissues, including the liver, kidney, brain, joints, and skin, emphasizing their property of preferential accumulation at pathological sites [152]. Huang et al. [44] modified two WYGRGL peptides (WL) onto the tetrahedral framework nucleic acid (tFNA) surface via click chemistry (tFNA-2WL) and loaded them with ginsenoside Rb1 (Gin) to form tFNA-2WL&Gin complexes (Figure 8c). In vivo fluorescence imaging showed that the drug-loaded complex tFNA-2WL&Gin maintained a strong signal within the joint seven hours post-injection, whereas free drug and unmodified drug-loaded tFNA were completely cleared within five hours. By using tissue clearing and light-sheet fluorescence microscopy, it was found that tFNA-2WL&Gin not only enriched in cartilage but was also widely distributed in key intra-articular structures such as the meniscus, joint capsule, and ligaments, achieving enrichment specifically within the lesion area.

## 6. Structure–Activity Relationships of Framework Nucleic Acid Composite Drugs

In drug delivery systems, FNAs offer distinct advantages for the ordered loading and precise delivery of drug molecules due to their unique structural features, including spatial programmability, high biocompatibility, and addressable modification sites [15,162]. A growing body of research indicates that the in vivo uptake efficiency, tissue distribution, and stability of FNA–drug composites are closely related to the size, geometry, and surface modification of the FNA. Rational geometric design not only enables fine control over drug loading behavior through modulation of base stacking density but also significantly influences receptor-mediated cellular uptake efficiency [163]. This chapter will first focus on size and shape effects, elucidating how different geometric configurations of FNAs regulate drug loading capacity and cellular uptake behavior. Subsequently, the effects of surface modification will be discussed, analyzing how the spatial distribution and density of targeting ligands or functional groups impact drug efficacy. Finally, from the perspective of linker design, the influence of linker length, rigidity, and cleavability on regulating drug release kinetics will be systematically analyzed, providing a structural design basis for constructing efficient and controllable FNA–drug delivery systems (Table 5).

### 6.1. Size and Shape Effects

FNAs are not only precise drug loading platforms, but their highly programmable geometry is also a core variable regulating their biological effects. Research shows that the geometry of FNAs, including their compactness, aspect ratio, rigidity, and overall dimensionality (2D or 3D), directly determines their stability in physiological environments, drug release kinetics, and cellular uptake efficiency.

The structural integrity of FNAs in complex physiological environments is a prerequisite for their function. Covalent linkage strategies for DNA nanostructures can significantly enhance their intracellular stability. Raniolo et al. [164] systematically compared the behavior of DNA nanostructures with covalent or non-covalent linkages during receptor-mediated endocytosis. They constructed five configurations: covalent and non-covalent tetrahedral (TD) and octahedral nanocages (OD), a rod-shaped chainmail (CM), as well as a non-covalent square-box (SBO) and rectangular origami (RO). The study showed that all structures were internalized primarily via the LOX-1 receptor, but geometry and linkage type significantly influenced uptake levels and intracellular fate. Compared to non-covalent origami structures, covalently linked TD and CM displayed higher cellular uptake, reaching 489 ± 107 and 541 ± 104 ng/106 cells within 3 h, respectively, and maintained structural integrity intracellularly for up to 18 h. This result indicates that covalent linkage can greatly enhance the intracellular stability of DNA nanostructures.

By precisely designing the geometry of DNA nanostructures, their resistance to enzymatic degradation can be effectively modulated at a physical level. Xu et al. [165] employed all-atom molecular dynamics (MDs) simulations to systematically elucidate, at the molecular level, the interaction mechanism of DNase I with double-stranded DNA of different sizes (15, 21, 26 bp) and with tetrahedral DNA nanostructures (TDNs). The study found that the binding of TDNs to the nuclease exhibits significant topology and size dependence: in 15 bp (TDN15) and 26 bp TDNs (TDN26), internal topological constraints induce an undertwist of approximately 32° in the edge DNA helices, leading to an increase in minor groove width, which sterically hinders the effective insertion of key residues of DNase I. Binding energy analysis confirmed that this structural protection mechanism, induced by conformational strain, results in significantly weaker binding energies for TDN15 and TDN26 compared to 21 bp TDNs (TDN21) and linear DNA of equivalent size.

**Figure 9:**
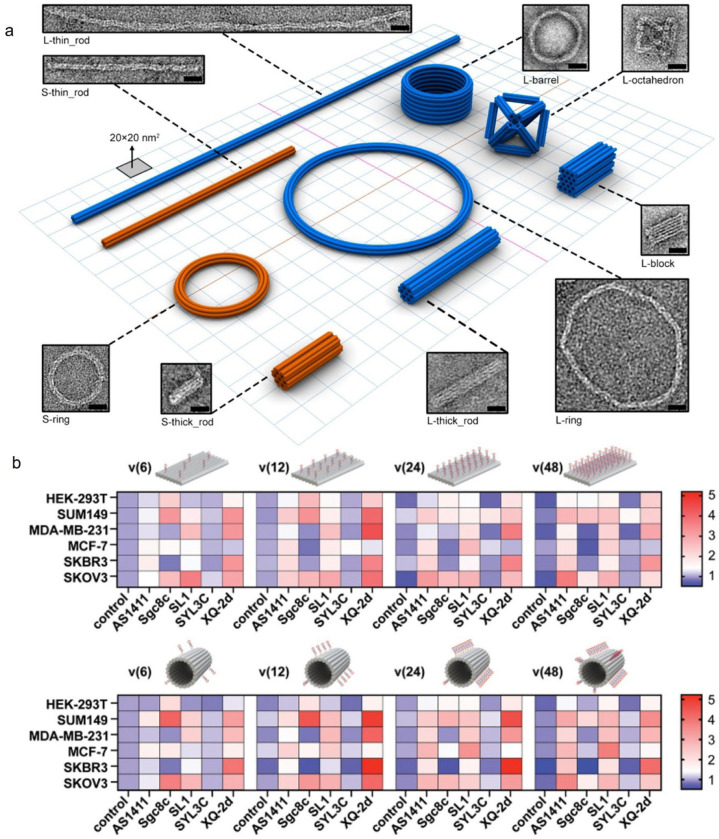
(**a**) DNA origami design with computer models and TEM images. Scale bars are 20 nm. Reprinted with permission from Ref. [166]. Copyright © 2018, American Chemical Society. (**b**) Heatmap of flow cytometric signals shows different cells binding with ^mono^mmvAp-DNA nanopatches (mvAp-DNPs, above) and ^mono^mvAp-DNA nanotubes (mvAp-DNTs, below) with various aptamers. Reprinted with permission from Ref. [167]. Copyright © 2024, American Chemical Society.

Building upon structural integrity, the shape, size, and structural compactness of FNAs are core determinants of their intracellular uptake efficiency. One group [61] demonstrated that FNAs can penetrate the epidermis only when the hydrodynamic diameter is ≤75 nm. Among these, the 21 bp small-sized tetrahedron (TH21, 17 nm) exhibited the best deep penetration performance (up to 350 μm) and, due to its compact structure, showed the strongest accumulation signal in skin fibroblasts. Bastings et al. [166] utilized DNA origami to construct 11 geometrically distinct DNA nanostructures (DNs), including circular, square, solid, and hollow shapes, demonstrating that nanocarriers with high compactness and low aspect ratio are generally more readily internalized by cells (Figure 9a). They found that regardless of being hollow or solid, more compact shapes were internalized first, with uptake efficiency showing a linear positive correlation with compactness (R^2^ = 0.84). DNA nanoparticles with a low aspect ratio (1–3) were most readily taken up by BMDCs (bone-marrow-derived dendritic cells). Under similar compactness, the intracellular concentration of solid L-block was significantly higher than that of hollow L-barrel (*p* < 0.05). Furthermore, the response to geometry varied significantly among different cell types; HUVE and HEK293 cells reached uptake saturation within 2 h, whereas BMDCs continuously internalized particles over 12 h, demonstrating the synergy between geometric effects and cell specificity.

Similarly, the rigidity and dimensionality of FNAs influence their intracellular uptake efficiency. Hu et al. [167] designed two DNA origami structures: DNA nanopatches (90 × 60 nm, DNPs) and DNA nanotubes (90 × 19 nm, DNTs), precisely controlling the valency and spatial arrangement of targeting aptamers (Figure 9b). The study found that the rigid tubular structure was more readily internalized by cells, achieving twice the uptake efficiency in MDA-MB-231 cells compared to the flexible sheet-like structure. By precisely arranging dual-targeting hetero-aptamers on the surface of the tubular carrier, they achieved ninefold higher specific uptake in tumor cells (MDA-MB-231) compared to normal cells. Furthermore, in the field of photothermal therapy, geometry plays a decisive role. Jiang et al. [168] constructed a DNA origami–gold nanorod (DO-GNR) composite by site-specifically assembling gold nanorods (GNRs) onto triangular and tubular DNA origami surfaces. They found that geometry was key to regulating cellular behavior: after 24 h of incubation, the triangular DO-GNR showed significantly better accumulation efficiency in human breast cancer MCF-7 cells than the tubular structure. Upon near-infrared laser irradiation (1.5 W/cm^2^), it more efficiently converted light energy into heat, thereby significantly inhibiting tumor growth.

In complex in vivo environments, the size and shape of FNAs collectively determine the tumor accumulation efficacy of delivered drugs. For drug delivery using 2D DNA nanostructures, triangular DNA origami possesses the most favorable passive tumor targeting ability and exhibits prolonged tumor uptake in vivo. Zhang et al. [71] systematically compared the effects of triangular, square, and tubular DNA origami on active tumor accumulation and drug delivery. The triangular origami, owing to its compact symmetric structure and uniform hydrodynamic size, demonstrated the best tumor accumulation behavior. This efficient accumulation, combined with the high drug loading capacity (Dox loading rate ~50%) afforded by the dense stacking of the triangular structure and its excellent acid-responsive release properties (drug release efficiency ~35% at pH 5.5 over 48 h, significantly higher than the 20% release under physiological conditions), significantly enhanced the in vivo antitumor effect and effectively alleviated the systemic toxicity induced by free Dox. Compact 3D DNA nanostructures also hold great potential for drug delivery due to their structural stability. Ijäs et al. [169] systematically compared the Dox loading and release behaviors of five DNA origami structures with different shapes and dimensions, including three 2D planar structures (triangle, bowtie, and double L-shape) and two 3D structures (DNA capsule and 24-helix bundle, 24HB). The results showed that under saturated loading conditions, the intercalation capacity for Dox differed minimally across configurations, with loading densities all around one Dox molecule per 2–3 base pairs. However, the more compact 3D origami, possessing higher helix density and overall rigidity, exhibited significantly enhanced structural stability in enzymatic environments. Specifically, the 24HB showed nearly two orders of magnitude greater resistance to DNase I degradation compared to the 2D triangle. Further release experiments revealed that this stability difference directly impacts Dox release behavior; the dense 3D structure significantly retarded drug release, whereas the 2D open structure was prone to faster release.

### 6.2. Functional Modification for Active Targeting

Surface modification of framework nucleic acid structures is a key strategy for achieving active targeting and improving biodistribution. By modifying specific DNA strand positions on FNAs with various functional groups or molecules, such as aptamers, small molecule ligands, peptides, and proteins, the responsiveness of FNA-based composite drugs to specific pathological environments and their therapeutic window can be significantly enhanced. In this process, the excellent addressability, high biocompatibility, and strong hydrophilicity of FNAs are fully leveraged.

Aptamers, as single-stranded oligonucleotides selected in vitro, offer high selectivity, low immunogenicity, and ease of synthesis, exhibiting high compatibility with DNA nanostructures. The relationship between an aptamer and its target cell is similar to a “lock-and-key” system; the affinity of a specific aptamer for a particular target effectively enhances the targeting ability of the modified construct, reduces non-specific drug distribution, thereby diminishing systemic toxicity, and allows for lower drug doses while enhancing efficacy [72,73,74,75,76,77,78,79,80,81,82]. Van Zundert et al. [170] employed single-particle tracking (SPT) to meticulously characterize the real-time interactions between DNA origami and cells in situ, quantitatively analyzing the diffusion behavior of DNA origami and performing multiparameter analysis of molecular binding interactions in the native cellular environment. Their results demonstrated that conjugation of specific aptamers to DNA origami structures conferred selectivity for EGFR-overexpressing MDA-MB-468 cells, and the binding time to the receptor was significantly longer than that of unmodified DNA origami.

Small molecule ligands are another common modification for FNAs. Compared to biomolecules like aptamers, their primary advantage lies in greater chemical stability and enhanced stability in complex biological environments. Among these, folic acid is one of the most extensively studied modifying molecules [85]. Specific folate receptors are overexpressed on the surface of various cancer cells, including ovarian and breast cancer [171], making folate a specific ligand for targeting these cancer cells and assisting FNAs in delivering loaded drugs to the target site. Bu et al. [172] precisely synthesized a folate-overhung mitoxantrone tetrahedral DNA framework. To verify in vivo efficacy, they established a leukemia cell xenograft mouse model. Furthermore, this work demonstrated, through data on tumor suppression, survival rate, body weight, and blood parameters, that the folate-modified mitoxantrone DNA tetrahedron exhibited superior performance compared to the unmodified DNA tetrahedron due to its targeting properties. Additionally, research has confirmed that peptide- and protein-based modifications can effectively enhance targeting to diseased cells, improve the accumulation of drugs carried by FNA nanocontainers at lesion sites, and enhance therapeutic effects [83,87].

### 6.3. Design of the Linker

FNAs can load drug molecules via linkers. In the design of FNA nanocontainers, a linker is typically a nucleic acid structure or small molecule with affinity for both the FNA and the drug to be loaded. Acting like a “crane arm,” the linker can be suspended from the FNA structure and serve as a bridge to load the drug onto the FNA. Linker design can be used to control the spatial arrangement of drug molecules and enable controlled drug release at the target site.

For nucleic acid-based linkers, their sequence composition and length influence the stability of the linker structure itself and its ability to load and release drugs. Danaeimoghaddam et al. [173] used coarse-grained oxidized DNA (oxDNA) and anisotropic network modeling (ANM) to study the stability and drug release kinetics of FNA nanocontainers (DNA origami in this work), using thrombin as a model drug. Systematic temperature-dependent simulations demonstrated that the length of the double-stranded linker affects the stability of the locking mechanism. Short linkers (3–4 base pairs) exhibited instability and a tendency to dissociate at 300 K, whereas long linkers (5–6 base pairs) showed good stability at 300 K, with significant dissociation observed only upon raising the temperature to 318 K or higher. The study also verified the impact of nucleic acid linker sequence design on its temperature stability. Pure A-T linkers showed significant transient mismatches at 318 K, which the authors attributed to the presence of only two hydrogen bonds per base pair and weaker stacking interactions. In contrast, C-G linkers showed no significant mismatches even at 323 K. Total hydrogen bond histograms indicated that mixed-sequence linkers possessed superior temperature stability compared to pure A-T or pure C-G linkers. Furthermore, in the 10-base pair loading system using thrombin as a model, mixed-sequence linkers exhibited longer drug release times compared to pure A-T linkers at all temperatures tested, with release times comparable to those of a 15-base pair loading system using a pure A-T linker.

Besides nucleic acid structures, small molecules like disulfide bonds are also used as linkers to load drugs onto FNA nanocontainers [174]. The disulfide bond is a covalent linkage that can be cleaved in response to the high expression of glutathione (GSH) in the tumor microenvironment. GSH concentrations in normal cells are typically insufficient to cleave disulfide bonds, thereby enabling drug release specifically within cancer cells [57].

**Table 5:** Key design parameters and principles of FNA-based drug delivery systems.

Key Design Parameter	Design Principle	Factor	Impact on Delivery Performance	Refs.
Size and shape effects	High compactness, low aspect ratio, high rigidity, 2D or 3D architecture selection based on requirement	Shape and compactness	Enhanced cellular internalization via high compactness and low aspect ratio	[166]
		Dimension	Superior rigidity and enzymatic stability in 3D structures, slower cargo release.	[167,169]
		Rigidity and flexibility	Higher uptake efficiency in rigid structures	[167]
Surface modification effects	Modification selection based on receptor type, precise control of modification spacing	Targeting ligand type	Precise lesion accumulation through receptor–ligand recognition	[170,171]
		Ligand density	Positive correlation between multivalent effects and uptake	[171,172]
Linker design	Stimuli-responsive moieties incorporation, linker length selection based on the desired release condition, enhance loading stability by increasing GC base content or using mixed sequences	Linker type	Site-specific, traceless release via stimuli-responsive linkers	[173]
		Linker length	Superior structural stability in 5–6 bp linkers over 3–4 bp segments	[173]
		Linker sequence composition	Superior thermal stability in mixed sequences over homopolymeric sequences	[174]

## 7. Summary and Outlook

### 7.1. Summary

As an emerging and progressively developing drug delivery system, framework nucleic acids (FNAs) primarily leverage their excellent addressability, programmability, and biocompatibility to function as nanocontainers for drug loading. This review comprehensively summarizes the research progress of FNAs as nanocontainers for delivering heterogeneous molecular drugs, including small molecule chemotherapeutics, functional peptides, and protein macromolecules. From a structural design perspective, FNAs can achieve precise drug loading through various modes, including physical encapsulation, covalent conjugation, and spatial entrapment. At the pharmacodynamic level, FNAs significantly improve the solubility of hydrophobic small molecule drugs, enhance the metabolic stability of peptides and proteins in complex physiological environments through protective effects, and improve drug bioavailability via their unique cellular uptake pathways. Whether in precision cancer chemotherapy, targeted therapy for inflammatory diseases, or in promoting tissue repair and combating microbial infections, FNA-based drug delivery systems demonstrate distinct advantages over traditional nanocontainers.

### 7.2. Challenges

Despite encouraging results in laboratory settings, FNA-based delivery systems face several critical challenges that must be addressed for industrial and clinical application. Firstly, the difficulty and high cost of large-scale synthesis represent the primary obstacles on the path to commercialization. Currently, the cost of synthesizing high-purity DNA strands remains prohibitive, especially for DNA origami structures requiring hundreds of short staple strands, where the synthesis process is complex and yields are limited. Furthermore, laboratory-scale annealing assembly processes, when scaled up to industrial levels, often encounter issues such as increased structural heterogeneity, decreased assembly efficiency, and difficulties in balancing efficiency and purity with purification methods such as ultracentrifugation. Secondly, limitations in drug loading capacity constrain pharmacodynamic output. Due to the inherently high-molecular-weight of the DNA framework, the mass ratio of carrier to cargo is often suboptimal. For disease scenarios requiring high-dose drug administration, insufficient drug loading often necessitates patients receiving high doses of nucleic acids, which not only increases treatment costs but also poses potential metabolic burdens. Additionally, potential immunogenicity and biosafety concerns cannot be overlooked. Although DNA is considered an endogenous biomolecule, FNAs used as nanocontainers in clinical therapy would still be perceived as exogenously introduced, high concentrations of nucleic acids. This could trigger recognition by pattern recognition receptors of the immune system, potentially inducing unintended immune–inflammatory responses. Furthermore, the excretion pathways of degradation products of FNAs and whether they induce anti-DNA antibodies lack support from large-scale, long-term preclinical studies. Although the stability of some FNAs, such as DNA tetrahedron, in complex physiological environments has proven sufficient for targeted delivery tasks, low concentrations of cations in the blood, such as magnesium deficiency, might lead to the dissociation of some framework structures that depend on cations for stability, causing premature drug leakage, which could reduce the precision of targeted delivery.

### 7.3. Outlook

To address the aforementioned challenges, future research can accelerate the development of FNA delivery systems through multiple approaches. In preparation, strategies such as phage amplification or rolling circle amplification (RCA) can reduce the cost of nucleic acid materials. For structural optimization, chemical modification of nucleotides or leveraging experiences from liposomes and viral vectors, such as polyethylene glycol (PEG) conjugation, lipid bilayer encapsulation, or biomimetic cell membrane modification, can protect immunogenic sites on the nucleic acid scaffold, potentially enhancing in vivo stability and prolonging the circulation duration of FNA delivery systems. In functional design, developing intelligent drug release systems with environmental-responsive properties represents a major trend in future drug delivery systems. By utilizing the strong addressability and programmability of FNAs, novel responsive switches incorporating mechanisms like pH response or glutathione concentration response can be developed for targeted cellular applications. Additionally, the synergistic integration of imaging molecules with therapeutic drugs can propel FNA systems toward integrated diagnostic and therapeutic platforms. In future research and development, multidisciplinary integration represents a promising pathway. By strengthening the deep integration of nucleic acid chemistry, materials science, immunology, and clinical medicine, pharmacokinetic and pharmacodynamic considerations can be incorporated at the design stage, promoting translational progress with the end goal in mind. This will help enhance the performance of FNA–drug delivery systems multi-dimensionally, from cost control to stability and intelligent responsiveness, propelling them as pioneers in nanomedicine to help humanity overcome challenges in the battle against diseases.

## Figures and Tables

**Table 1:** Heterogeneous molecular drugs loaded into FNA nanocontainers.

Category			Example(s)	Mechanism	Challenge(s)
Small molecule drugs	Anticancer	Anthracycline drugs	Doxorubicin and its derivatives or analogs	DNA base pair insertion	Systemic toxicity, drug resistance
		Inhibitor drugs	Paclitaxel, BKM120, shikonin	Inhibition of mitosis/enzyme activity	Low solubility, systemic toxicity, drug resistance
		Metal complex drugs	Pt(II) complexes, Pt(IV) complexes, Pt nanoparticles	DNA structural crosslinking and reactive oxygen species generation	Systemic toxicity, drug resistance
			Ruthenium polypyridine complex	Reactive oxygen species generation	Low solubility, low penetration ability
		Antimetabolite drugs	Gemcitabine	Inhibition of DNA synthesis	Drug resistance, short retention time
		Photosensitizer drugs	Heterocycle-based photosensitizer, anthocyanin	Reactive oxygen species generation	Low solubility, low penetration ability, poor stability
	Others	Quercetin, etc.		Different between each drug	Commonly low solubility, systemic toxicity
Peptide and protein drugs	Peptide	Antimicrobial peptides	GL13K, His-5	Bacterial cell membrane damage, reactive oxygen species generation	Systemic toxicity, enzyme degradation
		Antitumor peptides	KLA	mitochondrial membrane damage	Low penetration ability, enzyme degradation
		Pro-healing peptides	QKCMP	VEGF acceptor activation	Short retention time, non-specific distribution
	Protein	Enzymes	RNase A, glucose oxidase, horse radish peroxidase	RNA degradation, glucose consumption, reactive oxygen species generation	Short retention time, non-specific distribution
		Antibodies	Anti-VEGF/anti-human CD33/anti-human CDw328 antibody	Bind inhibition, immune response enhancement	Targeted toxicity; limited efficacy of single-target therapy
		Cytokines	Interleukin	Channel activation	Off-target effects

## Data Availability

No new data were created or analyzed in this study. Data sharing is not applicable.
